# Visual processing in the fly, from photoreceptors to behavior

**DOI:** 10.1093/genetics/iyad064

**Published:** 2023-05-02

**Authors:** Timothy A Currier, Michelle M Pang, Thomas R Clandinin

**Affiliations:** Department of Neurobiology, Stanford University School of Medicine, Stanford, CA 94305, USA; Department of Neurobiology, Stanford University School of Medicine, Stanford, CA 94305, USA; Department of Neurobiology, Stanford University School of Medicine, Stanford, CA 94305, USA

**Keywords:** neuroscience, vision, anatomy, physiology, computation, behavior, navigation, learning, FlyBook

## Abstract

Originally a genetic model organism, the experimental use of *Drosophila melanogaster* has grown to include quantitative behavioral analyses, sophisticated perturbations of neuronal function, and detailed sensory physiology. A highlight of these developments can be seen in the context of vision, where pioneering studies have uncovered fundamental and generalizable principles of sensory processing. Here we begin with an overview of vision-guided behaviors and common methods for probing visual circuits. We then outline the anatomy and physiology of brain regions involved in visual processing, beginning at the sensory periphery and ending with descending motor control. Areas of focus include contrast and motion detection in the optic lobe, circuits for visual feature selectivity, computations in support of spatial navigation, and contextual associative learning. Finally, we look to the future of fly visual neuroscience and discuss promising topics for further study.

## Introduction

Nervous systems evolved to allow animals to perceive, interact with, and move through the environment. In many animals, including humans and flies, vision is the dominant sensory modality. Vision is arguably best suited to perception at a distance, and its operation over short timescales enables dynamic guidance of ongoing behavior. In *Drosophila melanogaster*, each compound eye transmits information about the visual scene to over 100,000 neurons in each *optic lobe*, with both optic lobes together accounting for more than half of the neurons in the adult brain (Raji and Potter 2021). This dramatic allotment of biological resources to *visual processing* suggests both that vision plays a central role in fly behavior and that a significant amount of computing power is required to extract behaviorally relevant features from visual environments.

This review will discuss nearly a century of work examining visual processing and visually guided behavior in the fruit fly. These studies have taught us a great deal about the circuits and computational mechanisms that support vision. Despite vast anatomical differences, insect and mammalian visual systems perform many of the same *computations*, from the detection of motion to calculations of animal position and heading direction (reviewed in Clark and Demb 2016; Green and Maimon 2018). Further, the stereotyped and well-described anatomy and synaptic connectivity of the fly visual system have facilitated cellular (and sometimes subcellular)-resolution dissections of visual computation. These mechanistic insights have generated concise models of computation that can be tested at the circuit and cellular level in other model systems. In this way, studies of physiology and behavior in flies have revealed fundamental principles of visual processing that can be found across the animal kingdom. Here we have focused on a broad review of the literature, with the goal of introducing those new to the field to the many contributions that have been made. However, current work accounts for less than a quarter of the visually responsive neurons—even with everything we have learned, the fly visual system has many mysteries left to explore.

### *Drosophila* behavior relies heavily on vision

Given the scale of neural processing power devoted to vision, it is not surprising that this sense guides, evokes, or otherwise supports a variety of ethologically relevant behaviors. Perhaps the simplest visual behavior is *phototaxis*—an innate drive to fly or walk toward (or away from) light (Carpenter 1905; Heisenberg and Buchner 1977; Miller *et al*. 1981). In flies, phototactic behavior has been used extensively to dissect phototransduction and the neural mechanisms underlying *spectral preferences* (Hadler 1964; Benzer 1967; Pak *et al*. 1969). Given the choice between colored and white light of the same intensity or between 2 lights of different colors, flies show preferences for green (∼485 nm) and near-UV (∼365 nm) wavelengths (Bertholf 1932; Schümperli 1973; Hu and Stark 1977; Fischbach 1979; Gao *et al*. 2008; Yamaguchi *et al*. 2010; Karuppudurai *et al*. 2014; Otsuna *et al*. 2014). Overall, UV light attracts flies most strongly, but becomes aversive at high intensity. Importantly, phototactic preference is also under circadian control, with UV light eliciting the strongest attraction during subjective daytime hours (Hu and Stark 1977; Lazopulo *et al*. 2019).

Flies use *optic flow*, the pattern of motion generated by a visual scene moving over the eye, to guide ongoing locomotion. The “optomotor response” describes the tendency for a fly to turn in the direction of visual motion, a behavior that has been a focus of intense study for decades (Kalmus 1943; Götz 1964; Reichardt and Wenking 1969; Götz and Wenking 1973; Heisenberg and Götz 1975; Reichardt and Poggio 1976; Heisenberg and Wolf 1979; Götz 1987; Wolf and Heisenberg 1990; Tammero *et al*. 2004; Maimon *et al*. 2008; Mronz and Lehmann 2008; Theobald *et al*. 2010; Schnell *et al*. 2014). This optomotor response is most often studied with a tethered preparation, where a fly orients itself relative to a visual panorama. During forward movement, optic flow moves from front to back across both eyes, while side-slip or turning causes optic flow patterns that differ between the eyes. As a result, differences in optic flow signals across the eyes can indicate that the fly has been displaced off course and cause the fly to make a compensatory turn in the direction of visual motion. Similarly, flies can control their forward flight or walking speed using front-to-back visual motion signals (Budick *et al*. 2007; Katsov and Clandinin 2008; Fry *et al*. 2009; Rohrseitz and Fry 2011; Reiser and Dickinson 2013; Silies *et al*. 2013; Fuller *et al*. 2014; Creamer *et al*. 2018). Together, these reflexive maneuvers allow a fly to maintain straight, stable movement trajectories while walking or flying. Importantly, in addition to these stabilizing reflexes, flies can also voluntarily initiate course-changing turns that increase optic flow and are separately controlled (Ferris *et al*. 2018; Fenk *et al*. 2021).

Flies also respond to *looming stimuli*: objects with retinal coverage that expands in all directions, such as approaching predators, obstacles, or landing sites. Visual loom in the dorsal visual field causes walking flies to freeze in place or, if the loom is very large or very fast, causes them to initiate take-off escape maneuvers (von Reyn *et al*. 2014; Wu *et al*. 2016; von Reyn *et al*. 2017; Ache *et al*. 2019a). In flight, visual expansion, particularly in the ventral visual field, evokes rapid evasive reorientations or landing responses (Tammero *et al*. 2004; Bender and Dickinson 2006, Reiser and Dickinson 2013; Muijres *et al*. 2014; Ache *et al*. 2019b). Flies will also sometimes walk backwards in response to looming objects that approach slowly (Bidaye *et al*. 2014; Sen *et al*. 2017). Collectively, loom responses illustrate the importance of vision-driven behaviors for survival, as they allow flies to escape predation and avoid detection or collision.

Flies can also associate a variety of visual cues with reward or punishment. Environmental features such as brightness and color can provide contextual information that a fly can pair with positive or negative feedback (Quinn *et al*. 1974; Liu *et al*. 1999; Aso *et al*. 2014b; Vogt *et al*. 2014, 2016). In flight simulator experiments, oriented visual patterns and objects with different sizes, shapes, colors, or brightnesses can be associated with aversive stimuli (Dill *et al*. 1995; Wolf *et al*. 1998; Tang and Guo 2001; Liu *et al*. 2006; Zhang *et al*. 2007; Wang *et al*. 2008; Pan *et al*. 2009; Solanki *et al*. 2015; Koenig *et al*. 2016). Flies also use visual features of the environment to triangulate specific locations, demonstrating visual place learning (Ofstad *et al*. 2011; Haberkern *et al*. 2019). Perhaps more impressively, flies can remember the location of specific visual objects without prior training (Neuser *et al*. 2008; Kuntz *et al.* 2017; Sun *et al*. 2017). These observations jointly illustrate the utility of a wide range of visual features in supporting learned behavior.

Visual features such as landmarks or locomotor guidance cues also form the basis of long-range navigational behaviors. The sun is a prominent visual feature in natural settings and, as such, plays an outsized role in directing behavior. As noted above, solar UV light is highly attractive to flies. Flies can also sense the *polarization* of sunlight and use it as an orienting cue, often aligning their locomotion with the angle of polarization (Wolf *et al*. 1980; Wernet *et al*. 2012; Weir and Dickinson 2012; Velez *et al*. 2014; Mathejczyk and Wernet 2020). Sunlight polarization is common in natural settings, providing a reference frame for determining travel direction (Warren *et al*. 2018). This role of the sun as a landmark can be seen in *menotactic* locomotion, defined as straight-line travel over long distances in which a visual landmark is kept at a constant, arbitrary angle. The orientation of the sun, as well the distribution of its polarization angles, can guide this behavior (Giraldo *et al*. 2018; Warren *et al*. 2018; Green *et al*. 2019).

Beyond the prominent spatial cues provided by the sun, individual visual objects can also direct locomotion. High-contrast, vertically oriented objects—potentially representing a distant tree or other desirable perch—can attract flying and walking *Drosophila* (Reichardt and Poggio 1976; Strauss and Heisenberg 1993; Maimon *et al*. 2008, Robie *et al*. 2010; Ache *et al*. 2019b; Linneweber *et al*. 2020). The specific shape of such objects modulates their attractiveness, with taller objects being most attractive and shorter objects eliciting aversive responses (Maimon *et al*. 2008). However, flies will investigate dark spots when they are paired with attractive olfactory stimuli or during courtship (van Breugel and Dickinson 2014; Kohatsu and Yamamoto 2015; Ribeiro *et al*. 2018; Hindmarsh Sten *et al*. 2021). Flies are even capable of estimating the size and distance of terrain features or moving objects based solely on visual cues (Cook 1980; Pick and Strauss 2005; Agrawal *et al*. 2014; Kohatsu and Yamamoto 2015; Coen *et al*. 2016; Triphan *et al*. 2016; Ribeiro *et al*. 2018). This ability supports a diverse set of behaviors, including the pursuit of conspecifics during courtship and the crossing of terrain gaps during terrestrial navigation.

Collectively, this suite of visual behaviors is diverse, and there are undoubtedly additional visual behaviors that have not yet been discovered. Nonetheless, visual processing circuits must be sufficiently complex to extract many salient visual features and to flexibly couple these cues to a wide range of behavioral outputs.

### Resources, tools, and techniques to probe visual circuits

The wealth of publicly available anatomical and genetic resources makes the fly an excellent model for studying visual processing. For *anatomy*, nearly comprehensive atlases of optic lobe cell types exist, alongside well-annotated connectome studies (Fischbach and Dittrich 1989; Morante and Desplan 2008; Takemura *et al*. 2013; Nern *et al*. 2015; Morimoto *et al*. 2020; Kind *et al*. 2021; Shinomiya *et al*. 2022). These resources have facilitated an unambiguous assignment of functional properties to particular cell types and revealed how synaptic connectivity can support fundamental visual computations. Single-cell RNA sequencing data are also available for many visual system cell types, providing genetic insights into the function of each neuron (Kurmangaliyev *et al*. 2020; Özel *et al*. 2021; Davis *et al*. 2020; Konstantinides *et al.* 2022). Together, these resources create a fertile ground for understanding the diverse functions of neurons involved in visual processing.

**Fig. 1.**
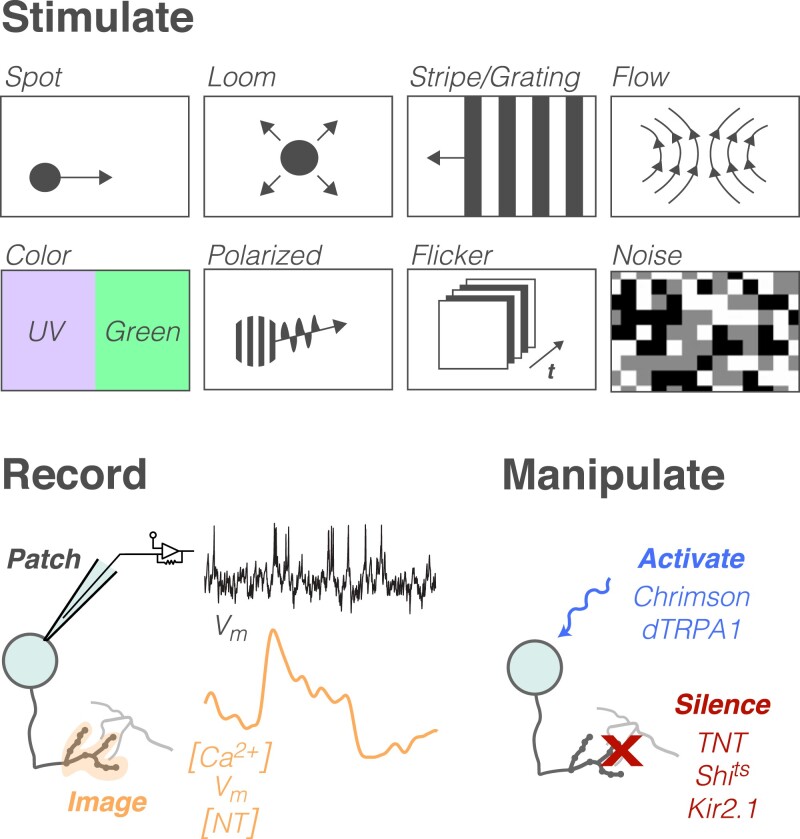
Tools and techniques to probe visual circuits. Top: illustrations of select visual stimuli. Bottom-left: recording methods. Membrane potential (*V*_m_) can be recorded via both patch clamp and imaging, while intracellular calcium concentration ([Ca^2+^]) and extracellular neurotransmitter concentration ([NT]) are most often measured with optical techniques. Bottom-right: common techniques for manipulating neuron function. See text for additional information about each tool.

How do researchers assess the *physiology* of neurons in the visual system? A typical study involves 3 components: visual stimuli that are presented to the fly, some means of monitoring the activity of neurons, and, potentially, perturbations of neuron or circuit function (Fig. 1). The stimulus set used to evoke responses is of critical importance, since stimulus design strongly shapes (and limits) neural responses. As a result, a wide range of stimuli have been devised to break down the complexity of natural scenes into simple components. For example, stationary and moving spots and bars, flicker, loom, and polarized and colored light have all been used to assess how the visual system computes (e.g. Joesch *et al*. 2008; Katsov and Clandinin 2008; Clark *et al*. 2011; Weir and Dickinson 2015; Heath *et al*. 2020; Sharkey *et al*. 2020; Hardcastle *et al*. 2021; Turner *et al*. 2022). Various kinds of noise stimuli have also been used to measure visual response properties (e.g. Clark *et al*. 2011; Behnia *et al*. 2014; Seelig and Jayaraman 2013; Leong *et al*. 2016; Li *et al*. 2021). A common preparation involves displaying stimuli to a restrained fly walking on a floating ball, which acts as a spherical treadmill. This entire setup is positioned under a microscope, allowing for the simultaneous collection of physiological and behavioral data (e.g. Seelig *et al*. 2010; Lyu *et al*. 2022). Under these conditions, visual feedback consistent with movement on the ball can be delivered, creating a so-called “closed loop” virtual reality, in 1D or 2D (e.g. Götz and Wenking 1973; Reichardt and Poggio 1976; Haberkern *et al*. 2019).

Monitoring (and manipulation) of neuronal activity requires *cell type specificity*—the ability to genetically target a neuron of interest—which can be elegantly achieved via binary expression systems, such as GAL4/UAS (Brand and Perrimon 1993). Massive libraries that tag neuronal subsets have been created, with GAL4 expression driven by particular enhancers or lineages (Pfeiffer *et al*. 2008; Gohl *et al.* 2011; Jenett *et al*. 2012; Silies *et al*. 2013; Awasaki *et al*. 2014). Specificity has been further refined by separating expression of the DNA binding and activating domains of GAL4 into 2 partially overlapping driver lines—by expressing each domain in a different line, only neurons labeled in both will show GAL4 activity (Luan *et al*. 2006; Dionne *et al*. 2018). These tools have facilitated extraordinarily precise targeting of specific cell types.

Once a driver line for a neuron of interest has been identified, *visually evoked activity* can be read out by monitoring *voltage fluctuations across a cell's membrane* (*V*_m_) or by observing how the *intracellular calcium* (Ca^2+^) concentration changes over time. *V*_m_ is most frequently assessed with whole-cell patch clamp or sharp electrode recording techniques (Joesch *et al*. 2008; Zheng *et al.* 2009), while Ca^2+^ is typically monitored with genetically encoded calcium indicators (GECIs) such as GCaMP and jRGECO (Chen *et al*. 2013; Dana *et al*. 2016; Zhang *et al.* 2023). More recently, *V*_m_ has also been recorded with genetically encoded voltage indicators (GEVIs) such as ASAP, Arclight, and JEDI (Yang *et al*. 2016; Tanaka and Clark 2020; Liu *et al*. 2022). As single-photon imaging generally interferes with visually evoked responses, *multiphoton imaging* is the method of choice for measuring Ca^2+^ or *V*_m_ dynamics. Moreover, recent work has developed sensors that can report *neurotransmitter release*, providing additional insights into visual processing (Marvin *et al*. 2018, 2019). Historically, all of these methods required a portion of the head cuticle to be dissected, exposing the brain; however, recent technical advances have raised the possibility of imaging through the intact head (Aragon *et al*. 2022). Selecting an appropriate recording method to monitor neuronal activity depends on practical considerations, such as the location and spatiotemporal selectivity of the neurons to be recorded or the desire to obtain simultaneous behavioral data.

Finally, researchers have taken advantage of the unique genetic resources available in *Drosophila* to precisely activate or silence genetically defined populations of visual system neurons or to disrupt gene expression. These experiments are frequently employed to determine the necessity or sufficiency of a specific gene or cell type for a particular visual computation or behavior. Tools for constitutively *silencing neurons* include expression of tetanus toxin light chain, which blocks synaptic release (Sweeney *et al*. 1995); expression of a mutant dynamin, encoded by *shibire*, which prevents vesicle recycling at certain temperatures (Grigliatti *et al*. 1973; van der Bliek and Meyerowitz 1991; Kitamoto 2001); expression of Kir2.1, which induces a potassium leak current, causing hyperpolarization (Paradis *et al*. 2001; Baines *et al*. 2001); or expression of GtACR, a chloride channel that depolarizes neurons exposed to green light (Mohammad *et al*. 2017). Each of these silencing tools has the ultimate effect of blocking synaptic release. Similarly, tools for *activating neurons* include TRPA1, a temperature-sensitive cation channel that depolarizes neurons warmed above room temperature (Hamada *et al*. 2008), and light-activated channelrhodopsin or Chrimson, which are cation channels that actively depolarize neurons only when they are exposed to blue or red light, respectively (Nagel *et al*. 2003; Inagaki *et al*. 2014; Klapoetke *et al*. 2014). Because flies can see blue light much better than red light, Chrimson has become the activation tool of choice. For the *inactivation of specific genes*, mutants, RNAi, FlpStop, and somatic CRISPR are all viable methods, and most of these techniques can be applied cell type specifically (Dietzl *et al*. 2007; Port *et al*. 2014; Xue *et al*. 2014; Port and Bullock 2016; Fisher *et al*. 2017; Port *et al*. 2020).

These methods for activating and silencing specific genes and neuronal populations are central to large-scale behavioral screens, which have long been used to uncover aspects of visual processing in the fly. In contrast to the targeted approach represented by the majority of physiological recording experiments, unbiased screens can also reveal how particular genes or neuronal populations influence phototactic or optomotor behaviors (e.g. Benzer 1967; Heisenberg 1972; Katsov and Clandinin 2008; Silies *et al*. 2013; Branson *et al*. 2009). More recent applications of this approach have even identified cell types involved in visually guided learning and social behavior (Aso *et al*. 2014b; Robie *et al*. 2017). Collectively, the tools and techniques for stimulating, recording, and manipulating neurons in the fly visual system are precise and sophisticated, facilitating a detailed description of visual function that is not currently possible in other model systems.

### Overview of the compound eye and visual system anatomy

**Fig. 2.**
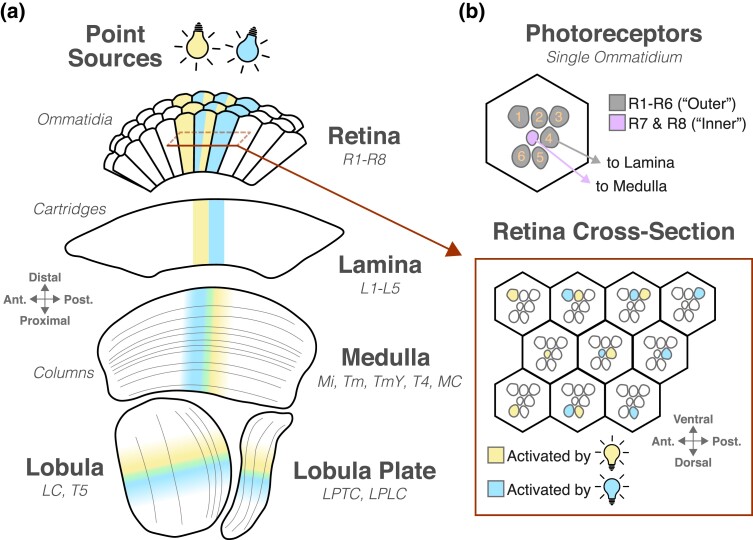
a) Overview of the compound eye and visual system anatomy. A simplified horizontal section through the optic lobe shows the organization of the early visual system. The portions of each neuropil that are activated by 2 adjacent point sources of light are highlighted in blue and yellow. Green indicates a mixing of signals from both sources. Red box indicates the location of the retinal cross-section shown in b). For regions with prominent laminar organization, layers are shown as thin gray lines. The primary feedforward cell types are listed for each neuropil. b) Top: a simplified cross-section through a single ommatidium shows the spatial arrangement of individual photoreceptors. Bottom: “Superposition” is illustrated in a cross-section through the retina, as indicated by the red box in a). The pattern of photoreceptors that respond to the blue and yellow point sources is shown.

Each compound eye contains ∼750 hexagonally arrayed facets called *ommatidia*, hexagonal structures that each collects light from about 5° of visual angle (see Fig. 2 inset; Heisenberg and Buchner 1977; Stavenga 2003). Light enters each ommatidium through the cornea and lens and is focused onto the *rhabdomere*, an anatomical specialization at the apical tip of each photoreceptor (Franceschini and Kirschfeld 1971; Zelhof *et al*. 2006). Six photoreceptors, designated R1–R6, are broadly responsive to UV and green light and have their rhabdomeres arranged around the outside of the ommatidium. Two photoreceptor classes, R7 and R8, are more narrowly tuned to particular wavelengths and stack their rhabdomeres at the center of the ommatidium (Heisenberg and Buchner 1977; Franceschini *et al.* 1981; Miller *et al*. 1981; Chou *et al*. 1996; Wernet *et al*. 2006; Takemura *et al*. 2008; Sharkey *et al*. 2020). In the dorsal-most part of the eye, called the *dorsal rim area*, R7 and R8 cells have rhabdomeres with specialized morphology (Wernet *et al*. 2003, 2012). This specialization results in each dorsal rim area photoreceptor responding to light with a specific polarization angle (Wernet *et al*. 2003; Weir *et al*. 2016). Across the dorsal rim area, the photoreceptor population as a whole can respond to light at any polarization angle.

The overarching organization of the fly visual system is *retinotopic*: each point in visual space is represented by a *column* of neurons, with neighboring points in space corresponding to neighboring columns. This anatomy is perhaps best understood by following the neural signals evoked by light emanating from a single point in visual space (Fig. 2a). This light depolarizes a particular spatial arrangement of photoreceptors that look at the same point in space and are distributed across nearby ommatidia (Fig. 2b; see below) (Vigier 1909; Kirschfeld 1967). These signals are then represented by 5 monopolar cells, L1–L5, in a single columnar unit within the *lamina*, the first neuropil in the optic lobe (Braitenberg 1967; Meinertzhagen and O’Neil 1991). In the lamina, these repeating units are called “cartridges” and house a highly stereotyped, almost crystalline anatomical and synaptic organization (Fischbach and Dittrich 1989; Meinertzhagen and O’Neil 1991; Takemura *et al*. 2008; Rivera-Alba *et al*. 2011). L1–L5 neurons then relay information from each cartridge to a column in the second optic lobe neuropil, the *medulla* (Fischbach and Dittrich 1989; Meinertzhagen and O’Neil 1991; Takemura *et al*. 2017). R7 and R8 inner photoreceptors that look at this same point in visual space synapse directly within the same column of medulla neurons (Fischbach and Dittrich 1989; Gao *et al*. 2008; Kind *et al*. 2021).

In the medulla, the spatial relationships between neighboring columns are preserved, but *lateral interactions* between columns are common (Fischbach and Dittrich 1989; Morante and Desplan 2008; Gao *et al*. 2008; Nern *et al*. 2015; Takemura *et al*. 2017; Kind *et al*. 2021). In addition, a distinct portion of the medulla specifically processes signals from the retinal dorsal rim area (Wernet *et al*. 2012; Weir *et al*. 2016; Kind *et al*. 2021). The medulla contains approximately 10 times more feedforward and laterally connected cell types relative to the lamina, reflecting a dramatic expansion in the complexity of visual processing (Fischbach and Dittrich 1989; Morante and Desplan 2008; Nern *et al*. 2015; Takemura *et al*. 2017; Kind *et al*. 2021; Shinomiya *et al*. 2022). The main feedforward neurons of the medulla project to the third optic lobe area, the lobula complex, and are primarily comprised of *transmedullary* (Tm and TmY) *cells*. In addition, the medulla is the most peripheral site of optic lobe output, with *medulla columnar* (MC) *neurons* projecting directly to the central brain (Li *et al*. 2020b; Yagi *et al*. 2016; Otsuna *et al*. 2014; Panser *et al*. 2016; Omoto *et al*. 2017; Timaeus *et al*. 2020).

Two discrete but densely interconnected neuropils comprise the *lobula complex*—the *lobula* and the *lobula plate* (Fischbach and Dittrich 1989; Morante and Desplan 2008; Shinomiya *et al*. 2019, 2022; Tanaka and Clark 2022a). While these regions receive significant retinotopic input from the medulla, the columnar segmentation of the lobula complex is less prominent than it is in the lamina and medulla. The lobula complex also provides the primary outputs of the optic lobe, with more than 30 classes of *lobula* and *lobula plate columnar* (LC and LPLC) neurons innervating a wide range of regions across the central brain (Otsuna and Ito 2006; Aso *et al*. 2014a; Vogt *et al*. 2016; Suver *et al*. 2016; Wu *et al*. 2016; Panser *et al*. 2016; Li *et al*. 2020a, b; Tanaka and Clark 2022a). In addition, many other morphologically distinct cell types have been shown to connect the lobula complex with the central brain (Otsuna and Ito 2006; Yagi *et al*. 2016; Li *et al*. 2020a). Finally, the lobula complex also represents a site in which signals from the 2 optic lobes are compared via direct morphological connections (Wu *et al*. 2016; Panser *et al*. 2016).

The next section will consider the visual computations that are performed in each of these ganglia.

## Optic lobes

In this section, we will discuss each of the major neuropils of the optic lobe, beginning peripherally with the retina and moving in *feedforward* fashion through the lamina, the medulla, and the lobula complex. For each neuropil, the goal of the text will be to synthesize our current understanding of its structure and function, including (1) circuit-level and cell type–specific anatomical features, (2) the physiological responses of well-studied cell types to visual stimuli, and (3) the behavioral effects induced by perturbations of well-studied cell types.

### The retina converts light into neural signals

#### Photoreceptor types express rhodopsin molecules with different wavelength sensitivities

**Fig. 3.**
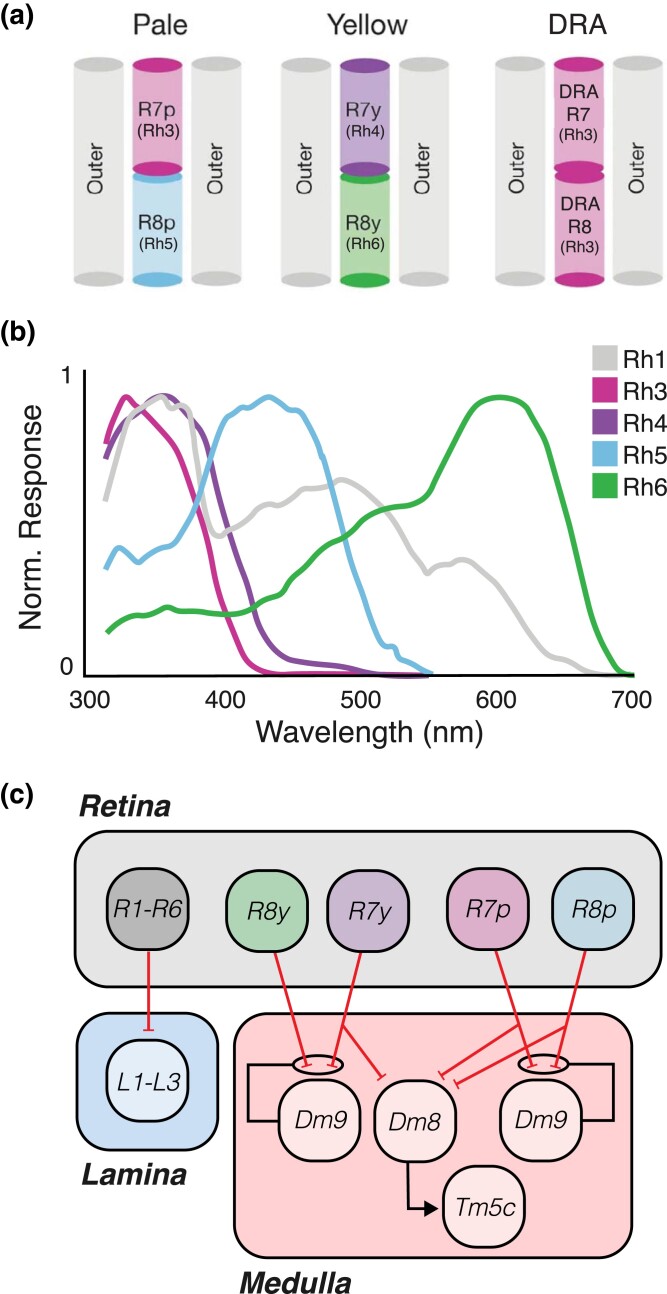
Photoreceptors transduce light of specific wavelength. a) Arrangement of photoreceptors in pale, yellow, and dorsal rim area ommatidia. In all cases, R1–R6 outer photoreceptors flank stacked R7 and R8 inner photoreceptors. The rhodopsin variants expressed in R7 and R8 determine ommatidium type. Adapted from Sharkey *et al*. (2020). b) Normalized photoreceptor responses by wavelength and opsin. Adapted from Sharkey *et al*. (2020). c) Downstream targets of photoreceptors. All photoreceptor types project from the retina (gray) and make inhibitory connections (red lines) in the lamina (blue) or medulla (pink). R7 and R8 segregate by ommatidium type and synapse onto Dm9 neurons in the medulla, which feedback presynaptically to mediate color opponency. Dm8 and Tm5c are known to mediate spectral preference behavior. Black lines indicate excitatory connections.

The retina is responsible for both detecting light and performing the initial stages of visual processing. The rhabdomeres of R1–R6 are arranged in a trapezoidal pattern around those of R7 and R8 (Fig. 2 inset), with R7 being superficial to R8 in the center of the ommatidium (Fig. 3a; Hardie 1985). R1–R6 cells differ from R7 and R8 in the *opsins*—light-sensitive molecules that initiate the phototransduction cascade—that they express. *R1*–*R6* express the rhodopsin Rh1, which is encoded by the *ninaE* gene (Scavarda *et al*. 1983; O’Tousa *et al*. 1985; Zuker *et al*. 1985). Rh1 is broadly sensitive to UV and blue–green light, with sensitivity peaks at ∼360 nm and ∼490 nm, reflecting both the spectral sensitivity of Rh1 itself and the presence of screening pigments that shield against longer wavelengths and sensitizing pigments that absorb and transfer energy from UV to the rhodopsin (Feiler *et al*. 1988; Sharkey *et al*. 2020). In contrast, each *R7* and *R8* cell generally expresses 1 of 4 different rhodopsin variants that are sensitive to different wavelengths (Fig. 3b). In some ommatidia, designated “pale,” R7 cells express Rh3 and are paired with R8 cells that express Rh5 (Fryxell and Meyerowitz 1987; Zuker *et al*. 1987; Chou *et al*. 1996; Papatsenko *et al*. 1997). Rh3 detects UV light with a peak at ∼330 nm, while Rh5 detects blue light with a peak at ∼435 nm in vivo (Feiler *et al*. 1992; Salcedo *et al*. 1999; Sharkey *et al*. 2020). In other ommatidia, designated “yellow,” R7 cells that express Rh4 are paired with R8 cells that express Rh6 (Rh4: Montell *et al*. 1987; Rh6: Huber *et al*. 1997). In vivo, Rh4 detects UV light with a peak at ∼355 nm, while Rh6 detects red light with a peak at ∼600 nm, a response that is shaped by the presence of an additional blue-absorbing yellow pigment that gives yellow ommatidia their name (Feiler *et al*. 1992; Salcedo *et al*. 1999; Sharkey *et al*. 2020). Pale and yellow ommatidia are distributed throughout the eye in a stochastic manner, forming a mosaic that varies between flies (Wernet *et al*. 2006). In dorsal rim area ommatidia, both R7 and R8 express Rh3 and have altered rhabdomere structures that align opsin molecules with specific polarization angles of light, with different ommatidia responding preferentially to specific polarization angles (Fortini and Rubin 1990; Wernet *et al*. 2003; Wernet *et al*. 2012). Finally, as flies age, R7 cells in ommatidia in the dorsal eye express both Rh3 and Rh4, broadening their spectral sensitivity (Mazzoni *et al*. 2008).

#### The phototransduction cascade depolarizes the photoreceptor

*Phototransduction* has been studied extensively in flies, leading to a detailed understanding of its molecular and cellular basis that we will summarize only briefly here (reviewed in Katz and Minke 2018). All fly photoreceptors depolarize in response to light (Wu and Pak 1975; Katz and Minke 2009; Hardie and Juusola 2015; Juusola and Song 2017; reviewed in Hardie and Juusola 2015). Photoreceptor rhodopsins are localized to the rhabdomere, a highly membranous structure composed of thousands of microvilli. Each microvillus contains all of the signaling molecules needed to convert absorption of a photon into a change in membrane potential via the phototransduction cascade. Rhodopsins are G protein–coupled receptors, and their activation initiates an intracellular signaling pathway, the phosphoinositide cascade. This cascade causes a phospholipase C enzyme, encoded by the gene *norpA*, to cleave a membrane phospholipid phosphatidylinositol-4,5-bisphosphate (PIP_2_). This cleavage triggers the opening of 2 specialized cation channels TRP and TRP-like (Niemeyer *et al*. 1996; Huang *et al*. 2004; Hardie and Franze 2012). TRP and TRP-like channels primarily conduct Ca^2+^ to depolarize the photoreceptor, resulting in synaptic transmission (Katz and Minke 2009; Hardie and Franze 2012; Hardie and Juusola 2015). Intriguingly, cleavage of PIP_2_ causes macroscopic displacements of rhabdomere membranes, thereby creating a photomechanical transduction mechanism (Hardie and Franze 2012; Juusola *et al*. 2017). This displacement creates a small, rapid shift in the viewing angle of the photoreceptor, thereby creating a “microsaccade” (Juusola *et al*. 2017; Kemppainen *et al*. 2022). These microsaccades, in turn, increase acuity for fast-moving objects, thereby improving sampling of motion signals (Kemppainen *et al*. 2022).

In dim light, the activation of 1 rhodopsin molecule by a single photon results in a small, discrete depolarization event, called a quantum bump (Wu and Pak 1975). Under dim illumination, the macroscopic response of photoreceptors can be thought of as the *linear* sum of many quantum bumps, meaning that the strength of photoreceptor responses scales linearly with light intensity. However, the visual system must operate across a large range of *luminance* levels, reflecting different light intensities. To do this, photoreceptors dynamically *adapt* their light sensitivity to avoid consuming excess energy or saturating their responses. As a result, photoreceptor responses become proportionally smaller, faster, and less noisy at higher luminance levels, and the dynamic range of photoreceptors also shifts to match stimulus statistics (Juusola and Hardie 2001; Nikolaev *et al*. 2009; Zheng *et al*. 2009; Juusola and Song 2017). Multiple mechanisms drive photoreceptor adaptation, including Ca^2+^-dependent regulation of phototransduction cascade components, inactivation of microvillar compartments, and *feedback* from downstream neurons (Nikolaev *et al*. 2009; Zheng *et al*. 2009; Song *et al*. 2012; Hardie and Juusola 2015; Juusola and Song 2017).

#### Photoreceptors feed into circuits that process motion, color, and polarization via histaminergic transmission

In all photoreceptor types, light-evoked potentials trigger the release of the neurotransmitter *histamine* (Pollack and Hofbauer 1991; Sarthy 1991). The enzyme histidine decarboxylase (Hdc) synthesizes histamine in photoreceptors, and *Hdc* mutants display normal light-evoked photoreceptor activity but lack downstream responses (Burg *et al*. 1993). Histamine acts as an inhibitory neurotransmitter by binding histamine-gated chloride channels encoded by the *ort* or *hisCl1* gene (Hardie 1989; Gengs *et al*. 2002; Witte *et al*. 2002). Ort is expressed in most postsynaptic targets of photoreceptors, while HisCl1 is expressed only in lamina glia and R7/R8 in the visual system (Pantazis *et al*. 2008; Tan *et al.* 2015; Schnaitmann *et al*. 2018).

Behavioral experiments reveal that R1–R6 contribute essential information to achromatic motion detection circuits, whereas R7 and R8 primarily contribute to circuits that process color cues and polarized light. Flies lacking R1–R6 cells display severe defects in the optomotor responses under most conditions (Heisenberg and Buchner 1977; Yamaguchi *et al*. 2008). However, flies can respond to optomotor stimuli without direct stimulation of R1–R6 by light, likely via gap junctions between the processes of R7 or R8 and R6 cells (Wardill *et al*. 2012). Conversely, R7 and R8 play central roles in mediating responses to light of different colors, and flies lacking R7 or R8 function display significant defects in spectral preference (Gao *et al*. 2008; Schnaitmann *et al*. 2018; Heath *et al*. 2020; Schnaitmann *et al*. 2020). Conversely, flies lacking R1–R6 function can discriminate between light of different colors, but R1–R6 can contribute to color vision in the absence of R8 (Schnaitmann *et al*. 2013). Finally, R7 and R8 in the dorsal rim area are both necessary and sufficient for behavioral responses to polarized skylight (Wernet *et al*. 2012).

#### Downstream visual signals guide retinal movements to allow active sensing

In spite of having a compound eye in which the ommatidial lenses are fixed in space relative to the fly's head, the fly is capable of eye movements that shift the retinal image (Fenk *et al*. 2022). To do this, musculature under the retinal can coordinately shift the positions of each rhabdomere across the visual field, resulting in a displaced image. These eye movements follow the direction of visual motion, are influenced by the spatial structure of the scene, and are actively engaged during both walking and flight, suggesting that they form part of an active sensing mechanism (Fenk *et al*. 2022).

### The lamina performs spatial and temporal contrast computations

#### Lamina neurons receive feedforward synaptic input from photoreceptors R1–R6

The axons of all photoreceptors from each ommatidium are bundled together and project directly into the lamina. Each bundle of axons is associated with a column of postsynaptic target neurons, creating a reiterated array of columns in the lamina that matches the number of ommatidia in the retina. The axons of R1–R6 photoreceptors terminate within the lamina, while those of R7 and R8 project through the lamina and into the medulla (Fig. 3c). As the rhabdomeres of R1–R6 cells are displaced from the central axis of the lens, each photoreceptor in the same ommatidium looks at a different point in space. To properly reconstruct an image, each cell must therefore project its axon to a different column of postsynaptic cells. Due to the curvature of the eye, this arrangement also means that R1–R6 cells from 6 different ommatidia look at the same point in visual space and converge onto the same column of postsynaptic cells. This combination of ommatidial optics and wiring comprises neural *superposition* and recreates a retinotopic map in the lamina (Fig. 2b; Vigier 1909; Trujillo-Cenoz 1965; Trujillo-Cenoz and Melamed 1966; Braitenberg 1967; Kirschfeld 1967; Kirschfeld 1973). By pooling inputs from multiple photoreceptors that collect light from the same point in space, neural superposition reduces noise, improving sensitivity under low-light conditions.

**Fig. 4.**
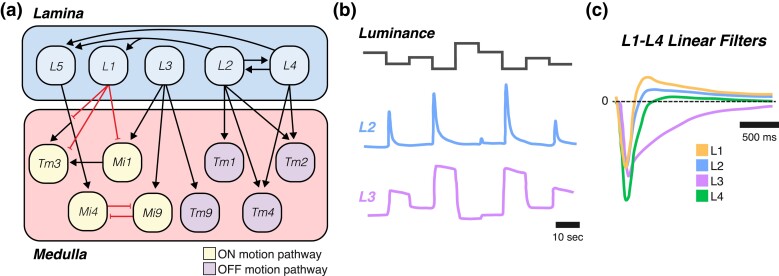
Contrast and luminance representation in the lamina. a) Wiring diagram of the ON motion (yellow) and OFF motion (purple) pathways in the lamina and medulla. ON/OFF here refers only to whether a neuron is upstream of the ON or OFF motion detectors (T4 or T5) and does not necessarily mean that the neuron itself is ON or OFF selective. Colors as in Fig. 3c. b) Schematic plots of L2 (blue) and L3 (purple) responses to changes in luminance. L2 responds only to decreases in luminance (OFF contrast), while L3 shows sustained OFF activity. Adapted from Ketkar *et al*. (2020). c) Temporal filters for L1–L4. L1 (orange), L2 (blue), and L4 (green) are biphasic and are therefore contrast selective. L3 (purple) is monophasic and is therefore luminance selective. Dashed line indicates the vertical position where filter strength is 0. Adapted from Clark *et al*. (2011) and Silies *et al*. (2013).

In addition to the photoreceptors, the lamina contains the processes of 12 types of neurons. Each column contains 5 types of *projection neurons* (L1–L5) that together represent all of the outputs of the lamina (Fig. 4a). In addition, the lamina contains 4 types of wide-field neurons (the *intrinsic amacrine neuron Lai*, the *tangential neuron Lat*, and the *wide-field neurons Lawf1/2*), and the feedback projections of 3 medulla cell types (the *centrifugal neurons C2* and *C3*, as well as *T1*) (Fischbach and Dittrich 1989; Meinertzhagen and O’Neil 1991; Hasegawa *et al*. 2011; Rivera-Alba *et al.* 2011; Tuthill *et al*. 2013).

Connections between lamina neurons can be feedforward or recurrent and may be confined to a single column or span multiple columns. R1–R6 photoreceptor cells make strong synaptic connections with L1, L2, and L3 (Fig. 3c), as well as weaker connections with Lai and glia through tetrad synapses in which 1 presynaptic R cell synapses onto 4 postsynaptic partners (Meinertzhagen and O’Neil 1991; Rivera-Alba *et al.* 2011). L1, L3, and L5 are solely postsynaptic in the lamina, but L2 and L4 are both presynaptic and postsynaptic (Fig. 4a). This arrangement facilitates recurrent signaling from L2 to R1–R6, L1, L4, and L5, as well as from L4 to R1–R6, L2, L5, and other L4 cells. Unlike other connections in the lamina that stay within the same point in visual space, these reciprocal connections between L2 and L4 can span neighboring cartridges (Meinertzhagen and O’Neil 1991; Rivera-Alba *et al.* 2011). The amacrine cell type Lai is also a prolific source of recurrent connections, forming pre- and postsynaptic connections with the majority of the other lamina cell types. The centrifugal cells C2 and C3 provide yet another set of feedback connections, in this case from the medulla, onto L1–L3 and L5. While not delineated here, the lamina wide-field neurons Lawf1/2 and the medulla neuron T1 form further synaptic connections within the lamina neuropil (Meinertzhagen and O’Neil 1991; Rivera-Alba *et al.* 2011; Tuthill *et al*. 2013).

#### Spatial receptive fields: L1–L5 respond to contrast in a spatially structured and cell type–specific manner

Lamina cells receive both direct and indirect inputs from photoreceptors. L1–L3 cells receive inhibitory histaminergic input from R1 to R6 and respond to input with graded potentials, hyperpolarizing in response to light increments (“ON” stimuli) and depolarizing in response to light decrements (“OFF” stimuli) (Nikolaev *et al*. 2009; Zheng *et al*. 2009; Yang *et al*. 2016; Ketkar *et al*. 2020). These changes in membrane potential drive decreases and increases, respectively, in intracellular calcium levels (Clark *et al*. 2011; Freifeld *et al*. 2013; Silies *et al*. 2013). *Calcium responses* in L4 resemble those of L2, whereas L5 responds oppositely, with calcium increases in response to ON stimuli and decreases in response to OFF stimuli (Silies *et al*. 2013; Meier *et al*. 2014; Drews *et al*. 2020; Matulis *et al*. 2020).

In addition to changing the sign of the photoreceptor response, lamina neuron responses have different spatial and temporal properties (Table 1). The *spatial receptive field* describes how strongly a neuron responds to stimulation from different points in visual space and can vary in size, shape, and polarity. Visual spatial receptive fields often have *antagonistic center-surround* organizations that form the basis of spatial contrast computations; that is, the same polarity of light (ON or OFF) will cause the cell to either depolarize or hyperpolarize depending on whether the stimulus is in the center or the edge of the receptive field (Kuffler 1953). L1–L4 all have OFF center, ON surround receptive fields, meaning the stimulus that would maximally depolarize these cells would be a small dark spot on a brighter background (Freifeld *et al*. 2013; Meier *et al*. 2014; Drews *et al*. 2020). Conversely, L5 has a center-surround receptive field with an ON center and an OFF surround (Drews *et al*. 2020). Moreover, the interactions between the center and surround of the receptive field can be complex—for example, in L2, the response to center stimulation alone and the response to surround stimulation alone do not linearly sum to the response to center and surround stimulation together (Freifeld *et al*. 2013).

**Table 1.** Response properties of retina and lamina neuron types.

Cell type	ON response	OFF response	Temporal properties	Feature selectivity	Role in motion detection	Neurotransmitter
*R1–R6*	+	−	Fast, monophasic	—	ON and OFF motion	Hist
*L1*	−	+	Fast, biphasic	Contrast and luminance	ON motion and some OFF motion	Glu
*L2*	−	+	Fast, biphasic	Contrast	OFF motion and some ON motion	ACh
*L3*	−	+	Slow, monophasic	Luminance	ON and OFF motion in low-light contexts	ACh
*L4*	−	+	Fast, biphasic	—	—	ACh
*L5*	+	−	Fast, biphasic	—	—	ACh

For each cell type, the response to ON and OFF stimuli is shown, along with known temporal response properties, feature selectivity, and neurotransmitter type. For ON and OFF responses, “+” indicates depolarization, while “−” indicates hyperpolarization. “Role in motion detection” refers to demonstrated behavioral effects. Hist, histamine; Glu, glutamate; ACh, acetylcholine.

#### Temporal filters: lamina neurons encode distinct time-varying features of visual stimuli

*Temporal receptive fields* describe how strongly a neuron responds to visual information from different points in time. These receptive fields are generally plotted as response strength as a function of time and can vary in kinetics, amplitude, and waveform. These plots are referred to interchangeably as *linear filters*, temporal filters, or linear kernels. If a neuron's temporal filter has a single positive lobe, it is described as “monophasic.” Conversely, if it has both a positive lobe and a negative lobe, it is described as “biphasic.” Biphasic filters typically suppress responses from farther back in time, and neurons with biphasic filters respond more strongly to stimuli that change in time relative to those that do not. Depending on the width of the lobes, these filters preferentially transmit visual information that changes over a particular range of timescales, a property referred to as “band-pass” or “high-pass” filtering. In contrast, monophasic filters integrate information over time, responding preferentially to sustained stimuli, and are sometimes referred to as “low-pass” filters.

L1, L2, L4, and L5 all have biphasic temporal filters that act over short timescales, meaning they tend to respond to stimuli that change more quickly (Fig. 4c; Clark *et al*. 2011; Silies *et al*. 2013; Drews *et al*. 2020; Matulis *et al*. 2020). L3, on the other hand, has a monophasic temporal filter (Silies *et al*. 2013). As a consequence, these cell types report different components of the visual stimulus over time: L3 encodes luminance, whereas L2 encodes *contrast*—the change in luminance (Fig. 4b and c; Ketkar *et al*. 2020). L1 responses are intermediate between L2 and L3, encoding both contrast and luminance (Ketkar *et al*. 2022). Intriguingly, the biphasic filters of L1 and L2 have different shapes in response to ON and OFF stimuli, revealing the dynamic nature of a neuron's temporal receptive field (Yang *et al*. 2016). L1 and L2 also alter their responses to repeated presentations of naturalistic contrast sequences to efficiently represent stimulus statistics (Nikolaev *et al*. 2009; Zheng *et al*. 2009). Additionally, L5 adapts strongly to the range of stimulus contrasts, adjusting their sensitivity as the contrast distribution changes (Matulis *et al*. 2020). Such findings suggest that lamina neuron properties may change dynamically in response to more complex or naturalistic stimuli.

While the visually evoked responses of most of the other lamina cell types have not been directly measured, 1 additional cell type, Lawf2, has been characterized in detail (Tuthill *et al*. 2014). Lawf2 responds to full-field ON flashes by depolarizing and spiking and responds selectively to low-frequency fluctuations in luminance. Intriguingly, the frequency tuning of Lawf2 is altered by flight and by *octopamine*, a neuromodulator associated with flight, suggesting that this neuron conveys information about behavioral state to the lamina and adjusts the gain of downstream responses to low-frequency inputs.

#### L1–L3 are critical for ON and OFF motion detection, whereas the function of other lamina cell types is less clear

L1–L3 have been classified as providing inputs to ON or OFF pathways by examining turning behavior and the responses of downstream neurons to stimuli that separately probe moving light or dark edges (*ON* or *OFF motion*). If silencing a cell type results in a different behavioral or neural response than in wild-type flies, then that cell type can be inferred to be critical in processing the type of motion stimulus that was tested. Such experiments initially suggested that L1 is required for normal responses to ON motion stimuli, and that L2 and L3 play important roles in responding to OFF motion stimuli (Joesch *et al*. 2010; Clark *et al*. 2011; Silies *et al*. 2013; Tuthill *et al*. 2013). However, experiments that explored a larger space of contrasts and adaptation states revealed that L1, L2, and L3 all contribute information critical to both the ON and OFF pathways, with L3 being particularly important in dim visual contexts (Ketkar *et al*. 2020; Ketkar *et al*. 2022). Studies have also rescued single L cell types by expressing ort in otherwise ort mutant flies, which have deficient L cell responses to photoreceptor input. When ort is rescued in L1, L2, or L3, motion processing is restored under some conditions, suggesting partial redundancy (Rister *et al*. 2007; Joesch *et al*. 2010; Ketkar *et al*. 2020, 2022). Silencing L4 led to divergent results, with some conditions showing no impact on ON or OFF responses and other conditions showing modest impairments to OFF motion detection (Silies *et al*. 2013; Tuthill *et al*. 2013; Meier *et al*. 2014). Finally, L5 has not been found to play a role in ON or OFF motion detection thus far (Tuthill *et al*. 2013). Table 1 summarizes the role of L1–L5 neurons in the ON and OFF motion pathways.

The role of the remaining neurons in the lamina is less clear. However, there is evidence that amacrine cells provide GABAergic lateral inhibition to monopolar cells and feedback inhibition to photoreceptors (Zheng *et al.* 2006; Nikolaev *et al*. 2009). In particular, perturbing synaptic transmission in Lai alters the recovery time of photoreceptors and L2, revealing that feedback and lateral connections alter the kinetics of visual responses (Hu *et al*. 2015; Wu *et al*. 2021). Similarly, while the visually evoked responses of T1 cells are unknown, silencing of T1 alters frequency-dependent orienting behaviors, suggesting that T1 also modulates temporal processing in L1 and L2 (Yuan *et al.* 2021). Finally, silencing Lawf2 cells also increases behavioral responses to relatively slow motion signals, consistent with this cell type subtracting low-frequency signals from inputs to motion processing (Tuthill *et al*. 2014).

### The medulla houses a diverse repertoire of cell types with distinct spatiotemporal properties

#### Medulla neurons relay information from L1–L5 and R7–R8 to the ON and OFF motion pathways

Relative to the lamina, the medulla contains a greatly expanded repertoire of cell types, with approximately 100 molecularly defined cell types, divided into at least 70 morphologically defined classes (Fischbach and Dittrich 1989; Strother *et al.* 2017; Özel *et al*. 2021). This diverse set of cell types is organized into a columnar array, reflecting ommatidial arrangement, and is stratified into 10 spatially segregated layers (designated M1–M10). Medulla cell types are classified into 5 morphological categories that include *medulla intrinsic* (Mi) *cells*, Tm cells, TmY cells, *distal medulla* (Dm) *interneurons*, and *proximal medulla* (Pm) *interneurons* (Fischbach and Dittrich 1989; Otsuna and Ito 2006; Morante and Desplan 2008; Nern *et al*. 2015; Kind *et al*. 2021). Each cell type typically receives input in 1 or more columns and provides synaptic outputs either within the medulla or in the lobula complex, with many arbors containing a mixture of pre- and postsynaptic elements. These medulla cell types receive input from L1–L5, R7, and R8, provide a dense network of connections among themselves, and feed back to the lamina via C2, C3, and possibly T1 neurons (Takemura *et al*. 2013; Shinomiya *et al*. 2014; Takemura *et al*. 2015, 2017; Shinomiya *et al*. 2019).

**Fig. 5.**
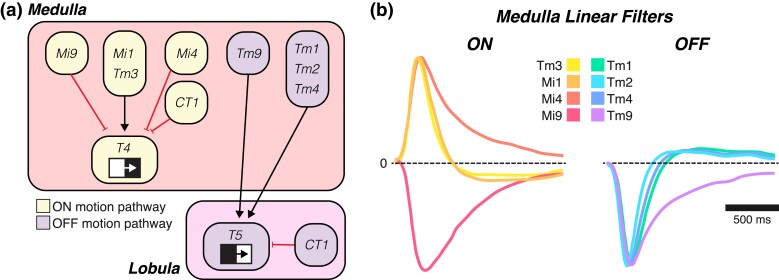
Inputs to the T4/T5 motion detector. a) Wiring diagram of the ON motion (yellow) and OFF motion (purple) pathways in the medulla and lobula. ON/OFF here refers only to whether a neuron is upstream of the ON or OFF motion detectors (T4 or T5) and does not necessarily mean that the neuron itself is ON or OFF selective. Each CT1 terminal functions independently, and the cell as a whole contributes to both ON and OFF motion. The spatial arrangement of inputs represents their relative anatomical positioning, with the leading edge on the left. b) Temporal filters for the ON (left) and OFF (right) motion pathways. Mi1 and Tm1–Tm4 are more biphasic, whereas Mi4, Mi9, and Tm9 are slower and more monophasic. Mi9 responds negatively to ON stimuli, unlike the rest of the ON motion pathway inputs. Plotting conventions as in Fig. 4c. Adapted from Arenz *et al*. (2017).

Although signals from L1, L2, and L3 contribute to both ON and OFF motion detection, they form synapses with distinct groups of cell types in the medulla (Fig. 4a). The main feedforward postsynaptic partners of L1 are Mi1 and Tm3; those of L2 are Tm1, Tm2, and Tm4; and those of L3 are Mi1, Mi9, Tm9, and Tm20 (Takemura *et al*. 2013; Shinomiya *et al*. 2014; Takemura *et al*. 2017; Shinomiya *et al*. 2019). L4 also forms some synapses with Tm2 and Tm4, and L5 receives input from L cells, especially L1, and feeds that back onto many L cells and medulla cells, such as Mi4. *Mi1*, *Mi4*, *Mi9*, and *Tm3* are the main presynaptic partners of T4 and will collectively be referred to as the “ON motion pathway.” *Tm1*, *Tm2*, *Tm4*, and *Tm9* are the main presynaptic partners of T5 and will collectively be referred to as the “OFF motion pathway” (Fig. 5a). T4 and T5 also have inputs from medulla neuron types that only indirectly receive information from L1 and L3, such as Mi4, CT1, and TmY15 (Takemura *et al*. 2017; Shinomiya *et al*. 2019).

#### Medulla neuron responses vary in ON/OFF selectivity and degree of rectification

As with cell types in the lamina, a fundamental method for understanding the physiology and function of medulla neurons has been to describe their spatial and temporal receptive fields. Like L1 and L2, many medulla neurons have antagonistic center-surround receptive fields. However, unlike L1 and L2, which respond to both ON and OFF stimuli (whether positively or negatively), many cell types in the medulla only depolarize or hyperpolarize in response to ON stimuli, responding much more weakly to OFF stimuli or vice versa. At its extreme, this unbalanced, *nonlinear* type of selectivity—strong responses to 1 polarity and no responses to the opposite polarity—has classically been referred to as “half-wave rectification.” In addition, *ON* and *OFF selectivity* can emerge as differences at the level of either membrane potential or intracellular calcium, suggesting that medulla circuitry can implement half-wave rectification using multiple mechanisms (Behnia *et al*. 2014; Yang *et al*. 2016; Kohn *et al*. 2021). As a central focus of the field has been on understanding motion detection, we will first describe medulla cell types that provide input to the ON motion detecting cell type, *T4*, then those that provide input to the OFF motion detecting cell type, *T5*. Finally, we will review the medulla cell types that have been linked to color vision and the detection of polarized light.

One function of the ON pathway is to produce selectivity for moving bright edges. However, the key inputs to T4 vary in their ON/OFF selectivity, as Mi1, Tm3, and Mi4 are ON selective, whereas Mi9 is OFF selective (Behnia *et al*. 2014; Strother *et al*. 2014; Yang *et al*. 2016; Arenz *et al*. 2017; Strother *et al*. 2017; Molina-Obando *et al*. 2019; Groschner *et al*. 2022). All of these cells have receptive fields that require integration across columns and display differences in the relative sizes of their centers and surrounds. In particular, Tm3 has a relatively large receptive field center compared with Mi1, Mi4, and Mi9. Additionally, Mi1 and Tm3 have little to no surround and respond well to both small and full-field stimuli. In contrast, Mi4 and Mi9 have antagonistic surrounds and are selective to small-field stimuli that approximate the size of their centers (Arenz *et al*. 2017; Strother *et al*. 2017). These 4 ON pathway neuron types also vary in their temporal receptive fields, with Mi1 and Tm3 having faster, biphasic linear filters and Mi4 and Mi9 having slower, monophasic linear filters (Fig. 5b; Arenz *et al*. 2017). Tm3 is slightly faster than Mi1 based on both voltage and calcium measurements (Behnia *et al*. 2014; Strother *et al*. 2017; Gonzalez-Suarez *et al*. 2022). Thus, Mi1 and Tm3 have band-pass properties and emphasize rapidly changing signals, while Mi4 and Mi9 have low-pass properties and are more integrative (Arenz *et al*. 2017). The response properties of ON pathway neurons in the medulla are summarized in Table 2.

**Table 2.** Response properties of medulla cell types.

Cell type	Temporal properties	Feature selectivity	Neurotransmitter	Output	Position of output
Mi1	Fast, biphasic	ON selective	ACh	T4	Center
Mi4	Slow, monophasic	ON selective	GABA	T4	Trailing edge
Mi9	Slow, monophasic	OFF selective	Glu	T4	Leading edge
Tm3	Fast, biphasic	ON selective	ACh	T4	Center
CT1 (med)	Fast, biphasic	ON selective	GABA	T4	Trailing edge
Tm1	Fast, biphasic	OFF selective	ACh	T5	Center
Tm2	Fast, biphasic	OFF selective (with ON information)	ACh	T5	Center
Tm4	Fast, biphasic	OFF selective	ACh	T5	Center
Tm9	Slow, monophasic	OFF selective (with ON information)	ACh	T5	Leading edge
CT1 (lob)	Fast, biphasic	OFF selective (with ON information)	GABA	T5	Trailing edge
Dm8	Slow, monophasic	UV–green color opponent	Glu	Tm5c	—
Dm9	Slow, biphasic	UV + green color selective	Glu	R7/R8	—

For each cell type, temporal response properties, visual feature selectivity, neurotransmitter, and major postsynaptic partners are shown. For neurons connected to T4 or T5, the position of that cell's output onto the T4 or T5 arbor is also listed (see Fig. 5a). CT1 has 2 entries because its neurites in the medulla (med) and lobula (lob) show distinct feature selectivity and connect with different postsynaptic partners. GABA, γ-aminobutyric acid; Glu, glutamate; ACh, acetylcholine.

One function of the OFF pathway is to produce selectivity for moving dark edges. The 4 key inputs to T5—Tm1, Tm2, Tm4, and Tm9—are all OFF selective and have antagonistic surrounds (Behnia *et al*. 2014; Strother *et al*. 2014; Meier *et al*. 2014; Fisher *et al*. 2015b; Yang *et al*. 2016; Serbe *et al*. 2016; Arenz *et al*. 2017; Kohn *et al*. 2021; Ramos-Traslosheros and Silies 2021). However, the degree of OFF selectivity varies across cell types, as Tm2, Tm9, and CT1 depolarize in response to OFF and hyperpolarize in response to ON, thereby retaining ON information within the OFF pathway (Meier *et al*. 2014; Ammer *et al*. 2015; Fisher *et al*. 2015b). Conversely, Tm1 and Tm4 are nonresponsive to ON stimuli under comparable conditions (Ramos-Traslosheros and Silies 2021). Interestingly, a subpopulation of Tm9 cells has a much larger receptive field center, with a diameter several times larger than those associated with Tm1, Tm2, Tm4, and nonwide-field Tm9 cells (Fisher *et al*. 2015b; Kohn *et al*. 2021; Ramos-Traslosheros and Silies 2021). For temporal response properties, Tm1, Tm2, and Tm4 fall into the fast biphasic category and function as band-pass filters, with Tm1 being slightly slower than Tm2 (Fig. 5b; Behnia *et al*. 2014; Serbe *et al*. 2016; Arenz *et al*. 2017; Yang *et al*. 2016; Kohn *et al*. 2021; Ramos-Traslosheros and Silies 2021). Tm9, in contrast, is slow and monophasic and therefore acts as a low-pass filter (Fisher *et al*. 2015b; Arenz *et al*. 2017; Kohn *et al*. 2021). The response properties of OFF pathway neurons in the medulla are summarized in Table 2.

*CT1* is an unusual cell type—in each optic lobe, a single CT1 cell extends a neurite into each of the columns in the medulla and lobula, providing input to every T4 and T5 cell, thereby contributing to both the ON and OFF motion pathways. Intriguingly, each columnar neurite acts as an independent processing unit with a small spatial receptive field and a biphasic temporal filter (Meier and Borst 2019). CT1 terminals in the medulla provide input to T4 and are ON selective, while CT1 terminals in the lobula provide input to T5 and are OFF selective (Meier and Borst 2019).

The spatial and temporal receptive field measurements we have described so far are snapshots of response properties that can vary dynamically. These adaptive processes can depend on the stimulus, the subcellular compartment being measured, the internal state of the animal, and whether voltage or calcium signals are being recorded (Zheng *et al*. 2009; Yang *et al*. 2016; Arenz *et al*. 2017; Strother *et al.* 2017; Drews *et al*. 2020; Ketkar *et al*. 2020; Matulis *et al*. 2020; Kohn *et al*. 2021). For example, some neuron types, like Mi1, can rapidly rescale their response amplitudes to match the dynamic range of the cell to the range of stimulus contrasts (Drews *et al*. 2020; Matulis *et al*. 2020). Moreover, while changes in membrane potential are relatively uniform across subcellular compartments, calcium responses can vary (Yang 2016). Finally, the neuromodulator octopamine, a signal indicative of the locomotor state of the animal, can accelerate and accentuate the biphasic temporal filters of specific medulla neurons, changes that tune these circuits to detecting faster motion signals (Chiappe *et al*. 2010; Maimon *et al*. 2010; Arenz *et al*. 2017; Strother *et al.* 2017; Kohn *et al*. 2021).

#### Overlapping roles for medulla neurons in the emergence of ON/OFF motion selectivity

Using both optomotor behavior and physiological measurements in direction selective neurons [T4, T5, and *lobula plate tangential cells* (*LPTCs*)], many studies have probed how silencing or activating individual medulla cell types, or pairs of cell types, influences motion processing. In the ON motion pathway, silencing Mi1 reduced T4, T5, and LPTC responses to moving ON edges at most stimulus velocities and contrasts and reduced optomotor turning. Silencing Tm3, on the other hand, had a more subtle effect, preferentially reducing responses to fast ON motion (Ammer *et al*. 2015; Strother *et al*. 2017). Silencing Mi4 or Mi9 had little impact on T4 responses but did increase behavioral responses to ON motion under some conditions, suggesting that these cells provide inhibitory inputs (Strother *et al*. 2017). Optogenetic activation of the 4 ON pathway medulla neuron types, both individually and in pairs, produced weak excitation of T4. However, simultaneous activation of Mi1 and Tm3 excited T4 more than expected from their separate contributions, suggesting that these 2 inputs are combined nonlinearly (Strother *et al*. 2017).

In the OFF motion pathway, silencing Tm2 and Tm9 produced the strongest reduction in responses to OFF motion, with more modest effects associated with silencing Tm1 and Tm4 (Meier *et al*. 2014; Fisher *et al*. 2015b; Serbe *et al*. 2016). In addition, combinatorial silencing generally increased these effects (Serbe 2016). Moreover, silencing Tm9 in combination with either L1 or L2 eliminated behavioral responses to OFF motion—much like the effect of silencing L3 with either L1 or L2 (Fisher *et al*. 2015b; Silies *et al*. 2013; Ketkar *et al*. 2020; Ketkar *et al*. 2022). These results suggest that Tm9 is an essential bridge between L3 and T5, and that Tm2 and Tm9 are key components of the OFF motion detection circuit.

Taken together, these studies identified important functions for some of the presynaptic inputs to T4 and T5, but suggest that there may be substantial overlap in functions for individual cell types. Extending these silencing and activation experiments into additional stimulus contexts may provide new insights into the functional overlaps. Finally, subtler perturbations of the temporal filtering properties of specific medulla cell types by targeting ion channels can further inform our understanding of the mechanisms underpinning direction selectivity (Gonzalez-Suarez *et al*. 2022).

#### Opponency contributes to color and polarized light processing in the medulla

To measure properties of light, such as color or polarization angle, requires that neurons compare the relative amplitudes of signals that have different spectral or polarization tuning. In neural systems, this computation often relies on opponency, defined as inhibitory interactions between input channels. The potential neural substrates of opponent comparisons begin at the level of *R7* and *R8* photoreceptors and include a diverse array of downstream targets across the medulla (Sancer *et al*. 2019; Sancer *et al*. 2020; Kind *et al*. 2021). Functional studies have demonstrated that reciprocal inhibitory connections between R7 and R8 cells within the same ommatidium create *color opponency* (Schnaitmann *et al*. 2018; Heath *et al*. 2020). Intriguingly, these opponent interactions can extend between neighboring ommatidia and are mediated by *Dm9* neurons (see Table 2 and Fig. 3c; Heath *et al*. 2020). These inter- and intraommatidial interactions construct a computationally efficient representation of chromatic content across the retina (Heath 2020). A second interneuron, *Dm8*, additionally integrates direct input from R7 and R8 with indirect input from R1 to R6. This integration creates spatially and chromatically opponent selectivity that accounts for UV–green phototactic preferences (Gao *et al*. 2008; Schnaitmann *et al*. 2013; Li *et al*. 2021; Pagni *et al*. 2021).

### The lobula complex performs directional motion computations

#### T4 and T5 receive spatially offset inputs in a precise manner

T4 and T5 neurons relay information from medulla neurons to the lobula plate, where they provide direction selective motion signals to many neuron types, including LPTCs and other visual projection neurons that connect the optic lobe to the central brain (Morimoto 2020; Klapoetke 2017; Boergens 2018). T4 receives inputs in the proximal medulla, whereas T5 receives input in the lobula. The axon terminals of T4 and T5 are organized into 4 layers within the lobula plate, each with different direction selectivity (Buchner 1984; Maisak 2013; Henning 2022; Yue 2016). This layer-specific axonal targeting pattern, combined with differences in gene expression, define 4 distinct T4 and T5 subtypes (Kurmangaliev *et al*. 2019). Finally, within each layer of the lobula plate, T4 and T5 terminals maintain the retinotopic arrangement of lamina and medulla columnar neurons, thereby creating a map of local motion signals across visual space.

T4 and T5 each receive inputs from presynaptic partners with receptive fields that are offset in visual space (Takemura *et al*. 2017; Shinomiya *et al*. 2019). The orientation of this offset aligns with the direction of motion each cell detects, meaning that a stimulus moving across the eye in a specific direction would sequentially cross the spatial receptive fields of a series of presynaptic partners (Fig. 5a). By convention, the presynaptic partner that is activated first is defined as on the “leading edge” of the T4/T5 receptive field, while the presynaptic partner that is activated last is defined as the “trailing edge.” T4 receives inputs on its leading edge from Mi9, in its center from Mi1 and Tm3, and on its trailing edge from Mi4, CT1, and C3. T5 receives inputs on its leading edge from Tm9, in its center from Tm1, Tm2, and Tm4, and on its trailing edge from CT1. Finally, there are also lateral connections among members of each T4 and T5 subtype (Takemura *et al*. 2017; Shinomiya *et al*. 2019).

#### Direction selectivity may involve both enhancing correct motion signals and suppressing incorrect signals

T4 and T5 have small spatial receptive fields and depolarize strongly to motion in a specific direction, referred to as the *preferred direction* (*PD*). At each point in space, each subtype of T4 (or T5) responds to a different preferred direction and relays this information to 1 of the 4 layers of the lobula plate (Maisak *et al*. 2013; Fisher *et al*. 2015a; Yue *et al*. 2016; Henning *et al*. 2022). However, the preferred directions represented by the 4 subtypes at each point in space vary across the visual field, reflecting the structure of the optic flow pattern produced by self-motion of the animal (Henning *et al*. 2022; Zhao *et al.* 2022). As a result, 1 of the layers of the lobula plate receives input from T4 and T5 cells selective for upward motion, 1 is selective for downward motion, and the other 2 layers pool cells that are selective for directions of motion that vary around the azimuthal plane (Buchner *et al*. 1984; Yue *et al*. 2016; Henning *et al*. 2022). While the dendrites of T4 and T5 are direction selective, the upstream medulla neurons are not, arguing that direction selectivity first emerges in the dendrites of T4 and T5 (Fisher *et al*. 2015a). Finally, T4 and T5 are also orientation selective and respond preferentially to static bars oriented orthogonally to the PD (Maisak *et al*. 2013; Fisher *et al*. 2015a).

The neural basis of direction selectivity has been investigated through behavioral experiments, computational modeling, and physiological measurements of T4 and T5. Such studies have drawn inspiration from classical *computational models* of elementary motion detection, such as the Hassenstein–Reichardt correlator, the Barlow–Levick model, and the motion energy model, which proposed minimal circuit or algorithmic structures that can produce direction selective outputs from nondirection selective inputs (reviewed in Yang and Clandinin 2018 and Ramos-Traslosheros *et al.* 2018; Hassenstein and Reichardt 1956; Barlow and Levick 1965; Adelson and Bergen 1985). At their core, these models reveal that any direction selective circuit must combine information from at least 2 spatially separated inputs. Moreover, each of these inputs must have different temporal filters, with 1 of the 2 inputs being slower and more monophasic than the other, causing the input from 1 point in space to be “delayed” relative to the other. As a result, if a moving stimulus reaches the point in space with the slower filter before it reaches the point in space with the faster filter, the differential temporal filtering will cause the 2 signals to reach a downstream circuit element at the same time. Conversely, if a moving stimulus reaches the point in space with the faster filter first, the differential temporal filtering will cause the 2 signals to reach a downstream circuit element at different times. The models differ in how a downstream element combines these 2 signals mathematically, either “adding” or “subtracting” the 2 signals linearly or “multiplying” or “dividing” the 2 signals nonlinearly. Depending on how the 2 points and the delay are arranged, the motion detector then generates either enhanced motion signals in the PD, or suppressed motion signals in the opposite direction [the “null direction” (ND)], or both.

These models make different predictions about how motion detecting circuits will respond to stimuli, and substantial effort in the field has gone into examining PD enhancement or ND suppression under different conditions. Overall, most studies have revealed that T4 and T5 appear to use similar computational mechanisms. However, depending on the measurement method or the stimulus used, different studies have found evidence for PD enhancement or ND suppression in both T4 and T5, suggesting that the neural circuit implementation of motion detection may be more complex or more flexible than the algorithmic architectures proposed in classical models (Clark *et al*. 2011; Tuthill *et al*. 2011; Maisak *et al*. 2013; Clark *et al*. 2014; Fisher *et al*. 2015a; Fitzgerald and Clark 2015; Haag *et al*. 2016; Leong *et al*. 2016; Leonhardt *et al*. 2016; Salazar-Gatzimas *et al*. 2016; Haag *et al*. 2017; Leonhardt *et al*. 2017; Strother *et al*. 2017; Gruntman *et al*. 2018; Salazar-Gatzimas *et al*. 2018; Wienecke *et al*. 2018; Gruntman *et al*. 2019; Agrochao *et al*. 2020; Gruntman *et al*. 2021; Kohn *et al*. 2021; Ramos-Traslosheros and Silies 2021; Groschner *et al*. 2022; Henning *et al*. 2022). In terms of measurement methods, T4 and T5 responses have been measured using electrophysiological recordings from cell bodies, voltage imaging from axon terminals, or calcium imaging from either dendrites or axons. In addition, a wide variety of stimuli have been used. These include structured stimuli like moving edges and drifting gratings, *noise stimuli*, minimal motion stimuli constructed by sequentially flashing 2 or spatially offset bars or spots, and nonmotion stimuli, like light flashes or static gratings. A variety of illusory motion stimuli, which can test specific nonintuitive predictions made by different models, have also been employed. Finally, experimental results have been complemented by an extensive body of computational modeling studies that have gone well beyond the classical models of motion detection (Eichner *et al*. 2011; Fitgzerald *et al*. 2011; Fitzgerald and Clark 2015; Leonhardt *et al*. 2016; Leonhardt *et al*. 2017). Remarkably, in spite of this extensive characterization, the mechanism by which T4 and T5 become direction selective remains under active investigation.

#### T4 and T5 cells play critical roles in many motion-sensitive circuit computations and behaviors

T4 and T5 are synaptically connected to many types of neurons in the lobula plate (Schnell *et al*. 2012; Boergens *et al*. 2018; Morimoto *et al*. 2020; Wei *et al*. 2020; Shinomiya *et al*. 2022). Of these, the best characterized are LPTCs, whose dendrites span large portions of the lobula plate and pool inputs from hundreds of T4 and T5 cells (Scott *et al*. 2002). Consistent with T4 and T5 providing significant input to LPTCs, blocking synaptic transmission in T4 and T5 substantially reduces both excitatory PD and inhibitory ND responses in LPTCs, without impacting responses to nonmotion stimuli (Schnell *et al*. 2012; Bahl *et al*. 2013). These effects are ON or OFF motion specific depending on whether T4 or T5 was silenced (Maisak *et al*. 2013). Furthermore, silencing T4 and T5 disrupts motion-evoked behavior over a range of speeds that suggests that additional processing may be needed to transform T4 and T5 outputs into behavior (Ammer *et al*. 2015; Strother *et al*. 2017; Creamer *et al*. 2018). More specifically, silencing T4 and T5 can prevent flies from following straight trajectories, both in flight and during walking (Cruz *et al*. 2021; Leonte *et al*. 2021). Finally, silencing T4 and T5 blocks behavioral responses to loom, and stimulation of T4 and T5 is sufficient to drive strong responses from at least 1 type of downstream loom detector, LPLC2 (Schilling and Borst 2015; Klapoetke *et al*. 2017). Collectively, these behavioral results are consistent with a role for T4 and T5 in providing inputs to many motion-dependent processes, including loom detection and course stabilization.

#### LPTCs represent wide-field motion signals and are modulated by locomotion

By pooling from specific T4 and T5 populations, LPTCs in fruit flies (and larger Diptera such as the blow fly *Calliphora*) become selective to particular patterns of optic flow, with prominent LPTCs preferentially responding to motion in the azimuthal plane (*HS cells*) or the vertical plane (*VS cells*) (Hausen 1984; Krapp *et al*. 1998; Joesch *et al*. 2008; Schnell *et al*. 2010). The precise directional tuning of each LPTC dendrite can have different tuning, creating a “matched filter” across the receptive field of the cell that is tightly aligned to the pattern of optic flow associated with a particular head movement. These exquisitely tuned filters emerge through the pooling of T4 and T5 signals from appropriate layers of the lobula plate, the variation in tuning of T4 and T5 across visual space, and nonlinear postsynaptic integration mechanisms (Scott *et al*. 2002; Barnhart *et al*. 2018; Henning *et al*. 2022). These cells also receive inhibitory signals from *lobula plate interneurons* (*Lpi cells*), which draw inputs from T4 and T5 cells with the opposite directional preference. Lpi inputs therefore create “motion opponency” in LPTCs (Mauss *et al*. 2017). In addition, LPTCs receive inputs containing substantial information about ongoing motor activity, including both efference copy and high-resolution walking speed signals (Kim *et al*. 2015; Fujiwara *et al*. 2017; Kim *et al.* 2017a; Fujiwara *et al*. 2022). Finally, LPTCs are coupled to specific populations of *descending neurons* (*DNs*), creating a pathway by which motion perception can be linked directly to behavioral modulation (Suver *et al*. 2016).

Many studies have explored the behavioral consequences of disrupting LPTC activity, with most perturbations focusing on HS cells. Bilaterally silencing or activating HS cells causes flies to reduce their translational velocity during walking, while having no effect on optomotor turning or wing steering during flight (Kim *et al*. 2015; Fujiwara *et al*. 2017; Busch *et al*. 2018). Conversely, unilaterally increasing HS activity promotes ipsilateral turning, while unilateral silencing promotes contralateral turning (Haikala *et al*. 2013; Fujiwara *et al*. 2017; Fujiwara *et al*. 2022). These results suggest that imbalanced HS activities between the 2 optic lobes, normally associated with rotational motion cues, are sufficient to drive turning. Thus, these data reveal the logic by which motion cues can promote turning and control walking speed and suggest that both HS and other cells play overlapping roles in guiding motion-dependent behavioral responses.

## Central brain

### Optic lobe signals are widely distributed across the central brain

**Fig. 6.**
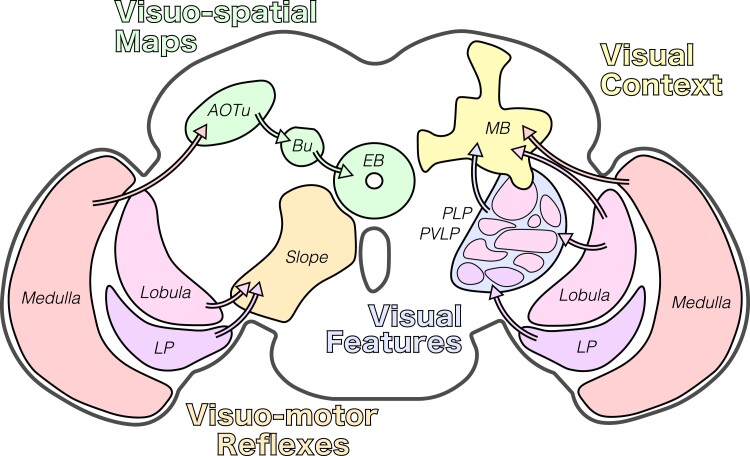
Optic lobe signals are widely distributed across the brain. A simplified coronal section through the fly brain is shown, with major vision-responsive neuropil drawn in different colors. Outputs from the optic lobe (medulla, lobula, and lobula plate) to 4 central brain structures are highlighted: the anterior visual pathway (green), the mushroom body (yellow), the optic glomeruli (blue), and the posterior slope (orange). The categories of visual information represented in each of these regions are indicated by colored text.

Four primary pathways carry visual signals out of the optic lobes and into the central brain (Otsuna *et al*. 2014; Aso *et al*. 2014a; Suver *et al*. 2016; Shiozaki and Kazama 2017; Omoto *et al*. 2017; Namiki *et al*. 2018; Li *et al*. 2020a; Timaeus *et al*. 2020; Li *et al*. 2020b; Hulse *et al*. 2021; Hardcastle *et al*. 2021). These pathways, which are comprised of *visual projection neurons*, “specialize” in particular visual signals (Fig. 6). While these specializations are not hard and fast, they are a useful way to conceptualize the distribution of visual information. For example, input to the *anterior visual pathway* and *central complex* is rigidly organized into retinotopic maps of visual space, ideal for guiding navigation. In contrast, visual input to the *mushroom body* tends to lack spatial information, instead carrying contextual signals that span large parts of visual space, such as ambient luminance. Specific visual features, such as object size and velocity, are extracted by visual projection neurons in the *optic glomeruli of the posterior lateral protocerebrum*. Finally, the optic flow signals that drive flight stabilization and course-corrective reflexes are delivered to descending circuits in the posterior slope.

#### LCs and LPLCs are selective for diverse and behaviorally relevant visual features

The most numerous connections between the optic lobe and the central brain are made by LCs and LPLCs projecting into the posterior lateral protocerebrum and posterior ventrolateral protocerebrum (Fischbach and Dittrich 1989; Otsuna and Ito 2006). These projections can be organized into approximately 20 dense synaptic clusters called “optic glomeruli” (Fig. 6). In an organization reminiscent of the antennal lobes, each LC or LPLC type selectively innervates a single glomerulus (Otsuna and Ito 2006; Mu *et al*. 2012; Wu *et al*. 2016; Panser *et al*. 2016). LCs and LPLCs collect feedforward input from columnar medulla projections and T neurons such as T4 and T5 (Klapoetke *et al*. 2017; Tanaka and Clark 2020; Keleş *et al*. 2020; Tanaka and Clark 2022a, b). Thus, visual projection neurons receive diverse signals from across the medulla and lobula complex, compressing rich retinotopic representations down to just tens of channels—a dramatic reduction that makes visual projection neurons likely candidates for higher-order visual feature selectivity (Mu *et al*. 2012; de Vries and Clandinin 2012; Panser *et al*. 2016; Wu *et al*. 2016).

**Fig. 7.**
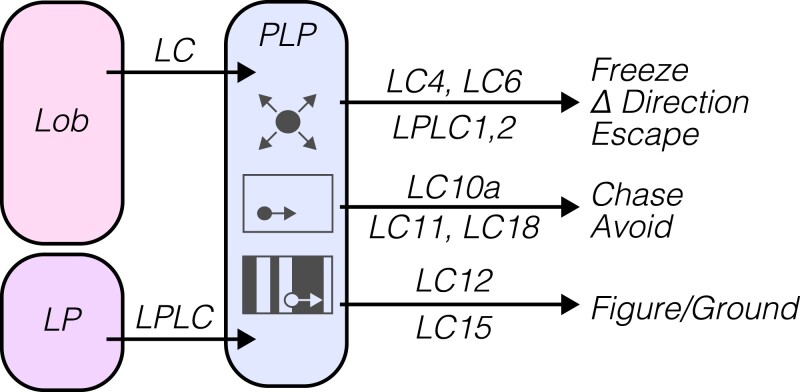
LCs and LPLCs are selective for diverse and behaviorally relevant visual features. A simplified illustration of lobula (Lob, pink) and lobula plate (LP, purple) inputs to the posterior (ventro-)lateral protocerebrum (PLP, blue) is shown. Select PLP visual representations are also schematized: loom (top), small moving objects (middle), and figure/ground discrimination (bottom). LC and LPLC types associated with each representation are indicated.

For some LC and LPLC types, different optic glomeruli appear to extract and relay visual features that serve distinct behavioral goals (Fig. 7; Wu *et al*. 2016; Klapoetke *et al*. 2022; Turner *et al*. 2022). In support of this observation, some LCs and LPLCs are directly upstream of descending circuits that control specific behaviors (Sen *et al*. 2017; von Reyn *et al*. 2017; Namiki *et al*. 2018; Ache *et al*. 2019a; Li *et al*. 2020b). One visual feature that has been well characterized in LCs and LPLCs is loom, the visual signature of an approaching predator or obstacle. In flies, these cues can elicit freezing, landing, and escape behaviors (von Reyn *et al*. 2014; Muijres *et al*. 2014; von Reyn *et al*. 2017; Sen *et al*. 2017; Ache *et al*. 2019b; Tanaka and Clark 2022b). Activation of *LC4*, *LC6*, *LPLC1*, and *LPLC2* all evoke freezing or take-off escape maneuvers, while activation of *LC16* can cause flies to walk backwards, another form of escape. Many of these visual projection neurons respond preferentially to looming visual objects, with LC6 spatially localizing loom sources via contralateral inhibition and LPLC2 deriving loom signals de novo by comparing local motion signals in a radial pattern (Klapoetke *et al*. 2017; Morimoto *et al*. 2020; Zhou *et al*. 2022). Finally, LPLC1, which promotes locomotor slowing and is tuned for object size and orientation, is recruited during object avoidance (Tanaka and Clark 2022b).

Many other LC types, including *LC10*, *LC11*, and *LC18*, have been implicated in the detection of small moving objects (Wu *et al*. 2016; Keleş and Frye 2017; Ribeiro *et al*. 2018; Tanaka and Clark 2020; Keleş *et al*. 2020; Städele *et al*. 2020; Hindmarsh Sten *et al*. 2021; Klapoetke *et al*. 2022; Turner *et al*. 2022). Small objects could indicate the presence of nearby conspecifics, or more distant predators, and therefore have ambiguous behavioral valence (Agrawal *et al*. 2014; Kohatsu and Yamamoto 2015; Tanaka and Clark 2020; Keleş *et al*. 2020; Hindmarsh Sten *et al*. 2021). Indeed, survival depends on correctly identifying and responding to the sources of these different visual cues. Consistent with this idea, different small object–detecting visual projection neuron populations have been linked to discrete behavioral goals. LC10a neurons—a subtype of LC10 that allows males to follow females during courtship—receive a dramatic boost in visual gain when copulation-promoting *P1* circuits are active (Kohatsu and Yamamoto 2015; Ribeiro *et al*. 2018; Hindmarsh Sten *et al*. 2021). When P1 circuits are not active, the female-tracking behavior evoked by LC10a cells is reduced, diminishing the likelihood of a male attempting to court an inappropriate target. LC11, which also tracks small moving objects, instead promotes freezing, potentially as part of a threat detection system (Keleş and Frye 2017; Tanaka and Clark 2020; Keleş *et al*. 2020). Finally, LC18 compares local contrast changes to identify motion at spatial scales much smaller than LC10 or LC11 (Klapoetke *et al*. 2022). Thus, while LC10, LC11, and LC18 are all small object detectors, the specific computations performed by each population are matched to different goals.

Recent studies have more comprehensively examined visual selectivity across many optic glomeruli (Klapoetke *et al*. 2022; Turner *et al*. 2022). Many LCs respond differently to visual objects on a stationary or moving background. This activity has been interpreted as figure-ground discrimination, but may also reflect a neural strategy to reduce the effects of self-motion blur (Aptekar *et al*. 2015; Keleş *et al*. 2020; Turner *et al*. 2022). Consistent with this latter effect, many LC responses are suppressed by background motion (Städele *et al*. 2020; Keleş *et al*. 2020). Conversely, in the presence of octopamine, a neuromodulator released during flight, some LCs become more sharply tuned (Städele *et al*. 2020). In particular, *LC12* and *LC15* gain selectivity for objects of different heights under these conditions, consistent with height having a strong influence on the relative attractiveness of visual objects (Maimon *et al*. 2008; Robie *et al*. 2010; Städele *et al*. 2020). The idea that octopamine and behavioral state might influence LCs is further supported by octopamine's gain-enhancing effects in other optic lobe circuits (Suver *et al*. 2012; Tuthill *et al*. 2014). Indeed, modulation of visual projection neuron responses is widespread, with unique contributions from motor efference and wide-field motion (Chiappe *et al*. 2010; Maimon *et al*. 2010; Kim *et al*. 2015, 2017a; Fujiwara *et al*. 2017; Städele *et al*. 2020; Fenk *et al*. 2021; Turner *et al*. 2022; Fujiwara *et al*. 2022). These results suggest that combinations of LCs are coregulated by behavioral state and may be jointly decoded by downstream circuits.

#### The anterior visual pathway carries visuo-spatial information to the central complex to guide navigation

**Fig. 8.**
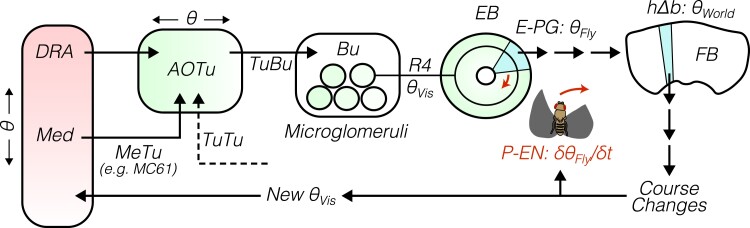
The anterior visual pathway (AVP) and coordinate transformations in the central complex. A simplified illustration of the anterior visual pathway is shown, with color gradients indicating different portions of visual space. Arrows indicate connections between neuropil, and the cell types that make some of these connections are noted. The anterior visual pathway relays a visual object's position in retinal coordinates (*θ*_Vis_), which are used to represent the fly's heading direction (*θ*_Fly_) as a bump of activity in E-PG neurons of the ellipsoid body (EB). When the fly turns, changes to *θ*_Fly_ (*δθ*_Fly_/*δt*) are represented in P-EN neurons, which rotate the E-PG activity bump. In the fan-shaped body (FB), *θ*_Fly_ is transformed into allocentric coordinates (*θ*_World_) in *h*Δ*b* neurons. See text for additional details. Med, medulla; DRA, dorsal rim area; AOTu, anterior optic tubercle; Bu, bulb.

The second major class of visual projection neurons, connecting the medulla and lobula to the *anterior optic tubercle*, represents the first step of the anterior visual pathway, which terminates in the central complex (Fig. 8). “MeTu” neurons—MC cells that project retinotopically to the anterior optic tubercle—form the bulk of this connection, with additional contributions from LCs (Otsuna and Ito 2006; Otsuna *et al*. 2014; Wu *et al*. 2016; Omoto *et al*. 2017; Sun *et al*. 2017; Ribeiro *et al*. 2018; Timaeus *et al*. 2020; Hulse *et al*. 2021; Hardcastle *et al*. 2021). The anterior optic tubercle is a highly segmented neuropil, with discrete domains innervated by multiple MeTu types representing different portions of visual space. For example, the anterior–posterior axis of the medulla is represented along a dorsal–ventral axis in the anterior optic tubercle, with a separate domain devoted to processing signals from the dorsal rim area (Omoto *et al*. 2017; Timaeus *et al*. 2020; Hardcastle *et al*. 2021; Hulse *et al*. 2021). The anterior optic tubercle projects directly to the *lateral accessory lobe* and the *bulb*, which are central complex accessory structures (Yang *et al*. 2013; Omoto *et al*. 2017; Sun *et al*. 2017; Timaeus *et al*. 2020; Hulse *et al*. 2021; Hardcastle *et al*. 2021). While the lateral accessory lobe is not retinotopically organized, the bulb is segmented into “microglomeruli” that represent different portions of visual space (Omoto *et al*. 2017; Sun *et al*. 2017; Shiozaki and Kazama 2017). These microglomeruli contain the dendrites of *ring* (R) *neurons*, which are integral components of the central complex, and the primary terminus of the anterior visual pathway.

The signals carried by the anterior visual pathway are thought to be important for navigation. As discussed in the *Introduction*, flies display a rich repertoire of navigation behaviors, and anterior visual pathway neurons respond to the visual cues that underlie each. For instance, the anterior visual pathway's preservation of spatial information is critical for a fly to determine the orientation or position of objects, cues that are needed to either approach an object or navigate relative to its position. In the dorsal bulb, “TuBu” and R neurons retinotopically encode object position in the ipsilateral visual field, and similar responses have been found in the anterior optic tubercle (Seelig and Jayaraman 2013; Omoto *et al*. 2017; Sun *et al*. 2017; Ribeiro *et al*. 2018; Hardcastle *et al*. 2021). Notably, bulb object representations persist after stimulus exposure, creating a short-term memory of object position (Neuser *et al*. 2008; Kuntz *et al.* 2017; Sun *et al*. 2017; Shiozaki and Kazama 2017). The positions of objects in the contralateral visual field are relayed by midline-crossing “TuTu” neurons to suppress ipsilateral anterior optic tubercle responses (Sun *et al*. 2017; Hardcastle *et al*. 2021). This positional opponency may allow flies to select between similar objects while choosing a navigational target (e.g. Haberkern *et al*. 2019).

Beyond object position tracking, the anterior visual pathway also carries many other signals important for navigation. For optic flow-driven maneuvers, the ventral domain of the bulb contains signals related to self-motion (Shiozaki and Kazama 2017). For color-based navigation, a subset of MeTu neuron types (*MC61*) is downstream of inner photoreceptors and is required for spectral preference (Gao *et al*. 2008; Otsuna *et al*. 2014; Timaeus *et al*. 2020; Hulse *et al*. 2021). Finally, skylight polarization-sensitive domains, downstream of the medulla dorsal rim area, are maintained throughout the anterior visual pathway, terminating at R4 ring neurons in the central complex (Omoto *et al*. 2018; Hardcastle *et al*. 2021). While the necessity and sufficiency of these anterior visual pathway signals have not been directly tested, the organization and feature selectivity of the anterior visual pathway strongly support its role in delivering navigation-relevant information to the central complex.

#### Color and luminance are visual context cues sent to the mushroom body

**Fig. 9.**
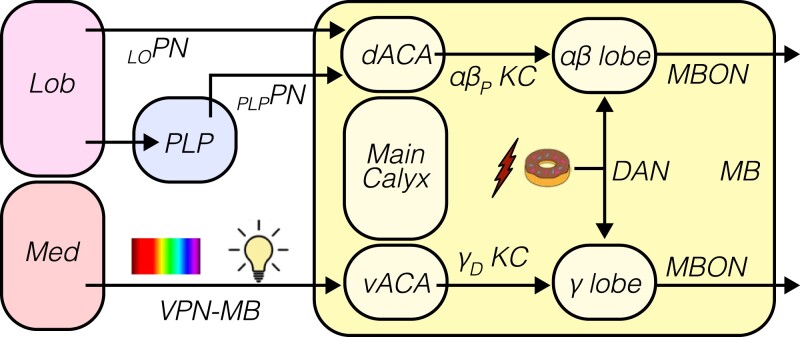
Color and luminance are contextual cues for visual learning in the mushroom body (MB, yellow). Lobula (pink) and medulla (red) inputs to the mushroom body are schematized, with arrows indicating connections between brain regions. Known cell types that form these connections are indicated. Medulla outputs carry information about the spectral content and brightness of ambient light to the ventral accessory calyx (vACA), while lobula outputs directly and indirectly connect to the dorsal accessory calyx (dACA). Visual inputs do not innervate the main calyx, where olfactory information enters the mushroom body. Distinct Kenyon cell (KC) populations carry information from the accessory calyces to mushroom body output neurons (MBONs), and dopaminergic neurons (DANs) modify the strength of this connection based on reward or punishment, facilitating associative learning.

A third major class of visual projection neuron relays information from the optic lobe to the calyx of the mushroom body, the associative learning center (de Belle and Heisenberg 1994). The calyx possesses multiple accessory structures, including the *dorsal* and *ventral accessory calyces*, which deliver visual information to the mushroom body circuit (Fig. 9). Both accessory calyces receive direct and indirect visual *projection neuron* input from the medulla and lobula, via *_LO_PNs* and *_PLP_PNs* (Vogt *et al*. 2016; Yagi *et al*. 2016; Li *et al*. 2020a, b). _PLP_PNs may also receive nonvisual inputs (Zheng *et al.* 2018; Li *et al*. 2020b), and some visual projection neurons innervate both the accessory calyces and the posterior lateral protocerebrum (Yagi *et al*. 2016). This circuit architecture suggests that _PLP_PNs play a feedforward role, while also integrating other signals. Two types of *Kenyon cells* (KCs), αβ_p_ and γ_d_, receive visual input in the dorsal and ventral accessory calyces, respectively. Importantly, these KC subtypes receive little, if any, input from the olfactory system and thus represent distinct mushroom body circuits specialized for the formation of visual associative memories (Aso *et al*. 2014a, b; Vogt *et al*. 2014, 2016; Yagi *et al*. 2016; Li *et al*. 2020b; Okray *et al*. 2022).

Visual projection neuron inputs to γ_d_ KCs in the ventral accessory calyx are comprised of independent channels conveying information related to distinct features of the environment, including the spectral content and ambient luminance (Vogt *et al*. 2016). Notably, visual information sent via visual projection neurons to the mushroom body has little spatiotemporal structure, in stark contrast to visual signals sent through the anterior visual pathway (Vogt *et al*. 2016; Li *et al*. 2020b). Thus, while optic glomeruli and the anterior visual pathway may represent spatiotemporal stimulus features important for navigation and other behaviors, mushroom body projections preferentially encode “context”—nonspatial features of the visual scene, such as ambient brightness and color, that are useful for associative learning (Aso *et al*. 2014b; Vogt *et al*. 2014, 2016; Okray *et al*. 2022). As such, the properties of visual projection neurons that project to the mushroom body are consistent with the overarching model that different visual projection neuron populations carry visual information that is matched to discrete behavioral goals.

### The central complex supports navigation and learning based on oriented visual landmarks

Visual signals from the anterior visual pathway converge on the central complex, a set of midline neuropils that have long been associated with navigation and are conserved across arthropod species (Loesel *et al*. 2002; Strauss and Heisenberg 1993). The central complex contains 4 primary regions—the *protocerebral bridge*, the *ellipsoid body*, the *fan-shaped body*, and a pair of *noduli*—as well as a larger set of accessory structures that include the anterior visual pathway's bulb and the DN-rich lateral accessory lobe (Hanesch *et al*. 1989; Yang *et al*. 2013; Wolff *et al*. 2015; Wolff and Rubin 2018; Franconville *et al*. 2018; Scheffer *et al*. 2020; Turner-Evans *et al*. 2020; Hulse *et al*. 2021). The protocerebral bridge, ellipsoid body, and fan-shaped body are organized into repeating columnar segments and discrete processing layers, reminiscent of the optic lobe. Visual input from the anterior visual pathway enters the central complex via R neurons that innervate the ellipsoid body, providing spatial maps of visual object position and self-motion (Seelig and Jayaraman 2013; Xie *et al*. 2017; Shiozaki and Kazama 2017; Omoto *et al*. 2017; Sun *et al*. 2017; Omoto *et al*. 2018; Fisher *et al*. 2019; Kim *et al*. 2019; Timaeus *et al*. 2020; Hardcastle *et al*. 2021). Additional visual input channels have been described anatomically, but have not been physiologically characterized (Hulse *et al*. 2021). For example, lateral accessory lobe inputs to the noduli are tuned for optic flow direction in bees and carry nonvisual orientation signals in flies (Stone *et al*. 2017; Currier *et al*. 2020; Hulse *et al*. 2021).

Across the central complex, different columns represent different portions of the visual environment oriented relative to the position of the fly's head (an “egocentric” visual representation; Seelig and Jayaraman 2015; Fisher *et al*. 2019; Kim *et al*. 2019; Shiozaki *et al*. 2020; Lyu *et al*. 2022; Lu *et al*. 2022). One population of columnar neurons, called *E-PGs*, is arranged in a ring and collectively encodes a fly's heading direction as a “bump” of increased activity in 1 location on the ring (Seelig and Jayaraman 2015). As the fly turns, the position of this bump rotates around the ring, providing an internal estimate of traveling direction (Fig. 8). This representation can be stable for many minutes in the absence of visual cues, but its stability and accuracy are greatly improved when the fly can see a landmark. This improvement in heading estimation depends on the visual activity of R neurons, which are required for landmark memory during navigation (Neuser *et al*. 2008; Kuntz *et al.* 2017; Fisher *et al*. 2019; Kim *et al*. 2019). Visual landmarks elicit R4 neuron-dependent suppression in E-PGs: as a fly moves through its environment, the visual scene changes, modifying the pattern of R neuron inhibition, allowing the E-PG compass to maintain a stable heading estimate across diverse scenes (Fisher *et al*. 2019; Kim *et al*. 2019; Turner-Evans *et al*. 2020). At the same time, self-motion signals associated with turning are passed to E-PGs by *P-ENs*, another class of central complex columnar neurons (Turner-Evans *et al*. 2017; Green *et al*. 2017; Su *et al*. 2017; Shiozaki *et al*. 2020). This P-EN input excites neighboring columns in the ellipsoid body, causing the E-PG bump to rotate around the ring, updating the internal heading estimate to account for recent turning. Local excitation from E-PG recurrence and P-ENs is offset by broad inhibition from another central complex cell type, Δ7 (Franconville *et al*. 2018; Turner-Evans *et al*. 2020). This combination of local recurrent excitation and broad inhibition is a hallmark of a specific computational model called a “ring attractor”; such models accurately predict central complex activity and fly turning behavior (Kim *et al*. 2017b; Kakaria and de Bivort 2017; Su *et al*. 2017; Turner-Evans *et al*. 2020; Vafidis *et al*. 2022).

The fan-shaped body uses the heading estimate generated in the ellipsoid body to perform additional computations in support of navigation. Fan-shaped body neurons are selective for diverse visual features—including small and large moving objects, visual expansion and contraction, and optic flow associated with free-flight maneuvers—with different fan-shaped body layers representing different features (Weir and Dickinson 2015; Shiozaki *et al*. 2020; Lyu *et al*. 2022; Lu *et al*. 2022). Fan-shaped body feature encoding is gated by flight, such that only minimal vision-evoked activity is seen in quiescent animals (Weir *et al.* 2014; Weir and Dickinson 2015; Currier *et al*. 2020). Different columnar cell types in the fan-shaped body (P-FNs) jointly encode egocentric heading direction and angular velocity as a pair of orthogonal vectors (Shiozaki *et al*. 2020; Lu *et al*. 2022; Lyu *et al*. 2022). In a computational process akin to vector multiplication, these multiplexed representations are combined with E-PG output to convert the fly's heading representation from egocentric to world-centered coordinates (Fig. 8). This coordinate-transformed representation of heading is encoded by another type of fan-shaped body columnar neurons called hΔb. Critically, hΔb activity scales with forward walking speed, meaning that it could be used by downstream circuits to compute how far the fly has walked, a form of path integration (Kim and Dickinson 2017; Stone *et al*. 2017; Vafidis *et al*. 2022). Finally, fan-shaped body representations are significantly less stable than the ellipsoid body compass, with rapid changes in P-FN tuning occurring in different visual environments (Shiozaki *et al*. 2020). Together, the ellipsoid and fan-shaped bodies provide a highly flexible network for contextualizing self-movement within the larger visual world.

The visual computations performed by central complex circuits are not universally recruited to facilitate every navigation behavior. Many reflexive maneuvers, such as the optomotor response, do not require the ellipsoid body compass, although fan-shaped body outputs can modify optomotor gain (Giraldo *et al*. 2018; Akiba *et al*. 2020). Only 2 strategies for vision-based navigation are known to rely on the central complex: menotaxis and visuo-spatial learning. Menotaxis refers to straight-line navigation over long distances, achieved by holding a visual landmark at an arbitrary angle during locomotion (Giraldo *et al*. 2018). Flies preferentially use this navigational strategy when sun-like stimuli are present, such as high-elevation, small, bright spots or directionally polarized light (Giraldo *et al*. 2018; Warren *et al*. 2018). As menotaxis relies on an internal comparison of a fly's current and target orientation relative to the visual landmark, silencing the ellipsoid body prevents the animal from holding the target at an arbitrary angle (Giraldo *et al*. 2018; Green *et al*. 2019).

The central complex is also recruited for visuo-spatial learning, particularly for memories involving visual object orientation (Strauss and Heisenberg 1993; Liu *et al*. 2006; Wang *et al*. 2008; Neuser *et al*. 2008; Pan *et al*. 2009; Kuntz *et al.* 2017). R neuron inputs to the ellipsoid body represent visual object orientation for tens of seconds after the object disappears, a process that relies on nitric oxide signaling (Sun *et al*. 2017; Shiozaki and Kazama 2017; Kuntz *et al*. 2017; Fisher *et al*. 2019). Indeed, many behaviors involving object orientation memory require R neuron function. For example, cell type–specific genetic rescue of mutants that disrupt memory formation demonstrated that plasticity in R neurons is required for flies to approach vanished vertical bars and form memories associated with spatially localized visual patterns (Neuser *et al*. 2008; Pan *et al*. 2009). Other types of associative memory also depend on the ellipsoid body, including visual place learning, where a particular location, identified by a specific visual scene, is linked to reward or punishment (Ofstad *et al*. 2011; Haberkern *et al*. 2019). In the fan-shaped body, different layers have been proposed to support this same kind of visual pattern learning, suggesting that memories of oriented visual objects may require more extensive central complex engagement, perhaps by contextualizing egocentric information in allocentric coordinates (Liu *et al*. 2006; Wang *et al*. 2008; Lu *et al*. 2022; Lyu *et al*. 2022).

### The mushroom bodies associate visual scenes with reward or punishment

Additional classes of visual associative learning require mushroom body function. The mushroom body is critical for the formation of olfactory associative memories, but early experiments found that mushroom body function was not required for associative learning based on oriented visual patterns, now known to rely on the central complex (see above; de Belle and Heisenberg 1994; Wolf *et al*. 1998; Liu *et al*. 2006; Wang *et al*. 2008; Pan *et al*. 2009). Oriented visual pattern learning requires the fly to associate a pattern's spatial location with an aversive stimulus (Tully and Quinn 1985; Wolf *et al*. 1998). In contrast, in classical olfactory learning, it is the identity of an odor, not its spatial distribution, that drives learning. Thus, if visual cues were to be used in associative learning, as the visual inputs to the accessory calyces would suggest, they would likely be nonspatial, contextual features of the visual scene (Quinn *et al*. 1974; Vogt *et al*. 2016; Yagi *et al*. 2016; Li *et al*. 2020a, b). Indeed, the mushroom body is required when oriented pattern memories are generalized to different visual contexts, such as a change in ambient illumination (Liu *et al*. 1999). More broadly, associative learning of many visual features rely on the mushroom body, including ambient color or brightness, as well as object size, color, or brightness (Tang and Guo 2001; Zhang *et al*. 2007; Aso *et al*. 2014b; Vogt *et al*. 2016; Solanki *et al*. 2015).

Mushroom body circuits that handle visual signals are largely independent from those for olfactory signals, although the general network architecture is similar for both sensory modalities (Fig. 9; Aso *et al*. 2014a; Li *et al*. 2021b). *αβ_p_* and *γ_d_ KCs*, positioned in the dorsal and ventral accessory calyces, respectively, project into the αβ and γ lobes, where they connect with *mushroom body output neurons* (*MBONs*). *Dopaminergic neurons* (*DANs*) modulate the strength of individual KC-to-MBON connections based on recent reward or punishment (Cohn *et al*. 2015; Handler *et al*. 2019). MBONs then connect to descending circuits in areas like the lateral accessory lobe, potentially integrating mushroom body and central complex outputs to jointly drive behavior (Li *et al*. 2020b; Scalpen *et al*. 2021). Just as αβ_p_ and γ_d_ KCs receive little olfactory input, their corresponding MBONs and DANs are similarly selective for visual information (Aso *et al*. 2014b; Li *et al*. 2020b). Consistent with this organization, KC and MBON activity in the αβ and γ lobes and DAN activity in the αβ lobe are all required for visual associative learning (Aso *et al*. 2014b; Vogt *et al*. 2014, 2016; Koenig *et al*. 2016; Liu *et al*. 2016; Okray *et al*. 2022). Finally, KC-to-KC and KC-to-MBON gap junctions also contribute to visual associative learning (Liu *et al*. 2016).

Although circuits for visual and olfactory learning appear to be largely independent, it is intriguing that multisensory cues are often the most potent for associative memory formation (Guo and Guo 2005; Thiagarajan *et al*. 2022; Okray *et al*. 2022). How might crosstalk between visual and olfactory associative memory circuits occur? One possibility is that these channels are integrated by downstream circuits (Li *et al*. 2020b; Scalpen *et al*. 2021). However, integration may also occur within the mushroom body itself, as nominally vision-selective γ_d_ KCs display increased olfactory sensitivity following paired visuo-olfactory training (Okray *et al*. 2022). Consistent with this notion, there is significant crosstalk between MBONs, and there is behavioral evidence that specific DANs may be used for both visual and olfactory learning (Vogt *et al*. 2014). Visual and olfactory learning signals might even be combined at the level of ventral accessory calyx inputs, as _PLP_PNs are hypothesized to contain nonvisual signals (Yagi *et al*. 2016; Li *et al*. 2020a). Thus, the apparent anatomical independence of mushroom body learning networks may belie multisensory learning processes that are engaged in more complex natural environments.

### DNs link visual processing to behavior

#### Loom, escape, and the giant fiber neuron

DNs are a diverse population of approximately 1,100 cells that receive input in the brain and project into the thorax, where they synapse onto cells in the ventral nerve cord (Hsu and Bhandawat 2016; Namiki *et al*. 2018). Because DNs are the *only* neurons delivering information from the brain to wing and leg sensorimotor networks, they represent a critical *computational bottleneck*. Individual DNs may collect information from across the brain or may instead have presynaptic terminals focused in a very narrow region. However, most DNs fall into discrete regional clusters, with enrichment in areas like the lateral accessory lobe, the wedge, and the posterior slope. DNs receive visual input from a variety of sources, including direct optic lobe input from LCs, LPLCs, and MCs; central complex output neurons in the lateral accessory lobe; and MBONs downstream of αβ_p_ and γ_d_ KCs (von Reyn *et al*. 2017; Sen *et al*. 2017; Namiki *et al*. 2018; Ache *et al*. 2019a; Rayshubskiy *et al*. 2020; Li *et al*. 2020b; Hulse *et al*. 2021). Thus, DNs are ideally suited for linking high-level visual processing to appropriate behavioral outcomes.

This linkage is best understood for the evolutionarily conserved *giant fiber neuron* (*GF*), also called DNp01, which drives a number of escape behaviors (Bacon and Strausfeld 1986; Namiki *et al*. 2018). A single action potential in the GF causes quiescent flies to leap into the air and initiate flight, with differences in spike timing eliciting distinct escape maneuvers (von Reyn *et al*. 2014). These action potentials are evoked by stimuli associated with predators, including visual loom and puffs of air (Mu *et al*. 2014; von Reyn *et al*. 2014). Visual loom can be parameterized as a combination of its retinal coverage and expansion velocity (Hatsopoulos *et al*. 1995). Loom depolarizes GF dendrites in the posterior lateral protocerebrum, with faster expansion and larger spatial coverage eliciting larger depolarizations (von Reyn *et al*. 2014, 2017; Ache *et al*. 2019a). Information about the rate of expansion and spatial coverage is relayed to the GF via discrete visual projection neuron channels, with LC4 activity representing expansion velocity and LPLC2 activity signaling retinal coverage (von Reyn *et al*. 2017; Ache *et al*. 2019a; Klapoetke *et al*. 2017; Morimoto *et al*. 2020). Because the axons of these visual projection neurons are organized into glomeruli, they target distinct regions of the GF dendrite (Wu *et al*. 2016; Panser *et al*. 2016; Morimoto *et al*. 2020). Overall, the GF circuit provides a striking example of how individual visual features, encoded by distinct visual projection neuron populations, can be combined to drive an ethologically appropriate behavioral response.

#### Control of vision-guided locomotion

**Fig. 10.**
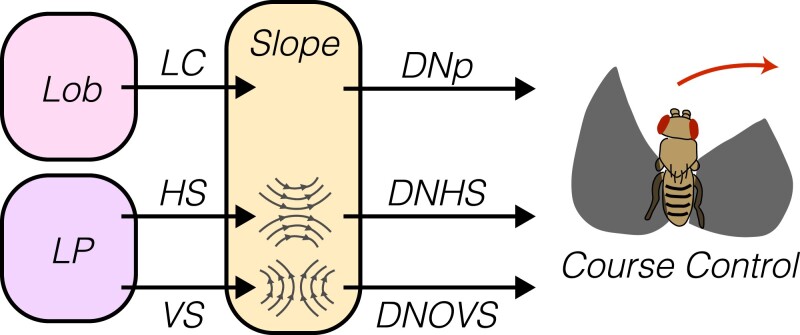
Descending neuron control of vision-guided locomotion. A simplified illustration of visual input to select DN populations is shown, with arrows indicating connections between brain regions. Known cell types that form these connections are indicated. Lobula plate (purple) outputs from the horizontal and vertical systems (HS and VS) carry information about wide-field optic flow to course-controlling DNs in the posterior slope (orange). Some lobula (pink) outputs also connect to DNs in this region.

Beyond the GF, a number of visually sensitive DN types can modulate ongoing walking or flying (Fig. 10). *DNOVS* and *DNHS cells*, which are downstream of wide-field motion detecting LPTCs, are tuned to optic flow along different rotational axes and are thought to facilitate flight stabilization maneuvers (Suver *et al*. 2016; Namiki *et al*. 2018). DNa02, which is downstream of fan-shaped body columnar cells (Li *et al*. 2020b), is engaged during ipsilateral, course-changing turns, but not course-correcting turns (Rayshubskiy *et al*. 2020). In contrast, a population code of *DNg02* neurons regulates wingbeat amplitude during optomotor course corrections (Namiki *et al*. 2022). Furthermore, a diverse set of loom-responsive DNs has been implicated in collision avoidance, with some evoking turns in flight (Schnell *et al*. 2017). Flies may also choose to land on an approaching object—*DNp07* and *DNp10*, which respond to front-to-back visual motion, cause flies to extend their legs in a stereotyped landing posture (Ache *et al*. 2019b). Walking flies must also avoid colliding with obstacles, and 2 types of DNs have been implicated in this behavior. The moonwalker descending neuron receives indirect input from the loom-sensitive visual projection neuron LC16 and causes the fly to walk backwards when activated (Bidaye *et al*. 2014; Wu *et al*. 2016; Sen *et al*. 2017). Similarly, DNp09 responds to visual loom and elicits freezing (Zacarias *et al*. 2018). Collectively, these studies suggest that different DN populations can modulate or trigger a variety of visually evoked locomotor maneuvers.

## Conclusion

Work in the fly has played a crucial role in expanding our understanding of the visuo-motor processing that occurs downstream of image-forming retinas. These studies have revealed circuit mechanisms for many essential visual computations, including the detection of directional motion and the transformation of information into different coordinate systems. These circuit and computational mechanisms have proven highly generalizable, closely paralleling work in vertebrate models, and the field has begun to define these mechanisms with a level of cellular and molecular precision that is challenging to reach in other systems (Clark and Demb 2016; Green and Maimon 2018). These advances, which lean heavily on the power and tractability of *Drosophila* genetics, highlight the utility of the fly in uncovering fundamental principles of visual processing. Moreover, these studies have laid the groundwork for 1 day defining a visuo-motor transformation all the way from retinal input to motor output, across an anatomically and functionally defined circuit.

These many successes have raised an even greater number of unanswered questions. For example, in the optic lobe, detailed physiological characterizations exist for only a fraction of the more than 100 anatomically identifiable cell types. As a result, it is unlikely that the known forms of feature selectivity—contrast detection, center-surround receptive fields, opponency, and so on—represent the full space of features extracted by early visual circuits. In the central brain, the link between visual processing and motor output has yet to be established for most stimuli and behaviors. For example, it remains unclear how heading direction signals and visual object position maps in the central complex dynamically recruit locomotor circuits in real time to guide how the animal actually moves. DNs represent a promising target for closing this gap because of their position as an anatomical bottleneck. More broadly, future work will likely uncover a broad space of DN-supported visual behaviors, as well as the organizational logic that links particular visual features to downstream motor programs. Connecting this organizational logic to the implementation of specific movements will require connecting DN input to the complex circuits of the ventral nerve cord, as well as understanding how ascending signals from the ventral nerve cord in turn affect visuomotor transformation. Finally, moving beyond this system’s neuroscience-level framework to an understanding of how molecules determine the computational function of each neuron and synapse remains a fundamental challenge.

The last 15 years has seen a remarkable acceleration of progress in *Drosophila* visual neuroscience. In this short time, the scope of our understanding has grown into a brain-wide tapestry of feature extraction, sophisticated computation, and sensory–motor loops. This rapid pace suggests the tantalizing possibility that a comprehensive understanding of fly vision may come sooner than expected. For the fly's eye, the future is bright.

## Data Availability

No data was generated as part of this manuscript.
